# Medicinal Plant Leaf Extract From Sage and Lemon Verbena Promotes Intestinal Immunity and Barrier Function in Gilthead Seabream (*Sparus aurata*)

**DOI:** 10.3389/fimmu.2021.670279

**Published:** 2021-05-14

**Authors:** Ricardo Salomón, Felipe E. Reyes-López, Lluis Tort, Joana P. Firmino, Carmen Sarasquete, Juan B. Ortiz-Delgado, José C. Quintela, José M. Pinilla-Rosas, Eva Vallejos-Vidal, Enric Gisbert

**Affiliations:** ^1^ Aquaculture Program, Institut de Recerca i Tecnologia Agroalimentàries (IRTA), Centre de Sant Carles de la Ràpita (IRTA-SCR), Sant Carles de la Ràpita, Spain; ^2^ PhD Program in Aquaculture, Universitat Autònoma de Barcelona, Bellaterra, Spain; ^3^ Department of Cell Biology, Physiology and Immunology, Universitat Autònoma de Barcelona, Bellaterra, Spain; ^4^ Facultad de Medicina Veterinaria y Agronomía, Universidad de Las Américas, Santiago, Chile; ^5^ Consorcio Tecnológico de Sanidad Acuícola, Ictio Biotechnologies S.A., Santiago, Chile; ^6^ Instituto de Ciencias Marinas de Andalucía (ICMAN-CSIC), Universidad de Cádiz, Cádiz, Spain; ^7^ Natac Biotech, Madrid, Spain; ^8^ Centro de Biotecnología Acuícola, Departamento de Biología, Facultad de Química y Biología, Universidad de Santiago de Chile, Santiago, Chile

**Keywords:** cell proliferation, feed additive, gut health, innate immunity, lectin histochemistry, ursolic acid, verbascoside acid, GALT

## Abstract

The inclusion of a medicinal plant leaf extract (MPLE) from sage (*Salvia officinalis*) and lemon verbena (*Lippia citriodora*), rich in verbascoside and triterpenic compounds like ursolic acid, was evaluated in gilthead seabream (*Sparus aurata*) fed a low fishmeal-based diet (48% crude protein, 17% crude fat, 21.7 MJ kg^-1^, 7% fishmeal, 15% fish oil) for 92 days. In particular, the study focused on the effect of these phytogenic compounds on the gut condition by analyzing the transcriptomic profiling (microarray analysis) and histological structure of the intestinal mucosa, as well as the histochemical properties of mucins stored in goblet cells. A total number of 506 differentially expressed genes (285 up- and 221 down-regulated) were found when comparing the transcriptomic profiling of the intestine from fish fed the control and MPLE diets. The gut transcripteractome revealed an expression profile that favored biological mechanisms associated to the 1) immune system, particularly involving T cell activation and differentiation, 2) gut integrity (i.e., adherens and tight junctions) and cellular proliferation, and 3) cellular proteolytic pathways. The histological analysis showed that the MPLE dietary supplementation promoted an increase in the number of intestinal goblet cells and modified the composition of mucins’ glycoproteins stored in goblet cells, with an increase in the staining intensity of neutral mucins, as well as in mucins rich in carboxylated and weakly sulfated glycoconjugates, particularly those rich in sialic acid residues. The integration of transcriptomic and histological results showed that the evaluated MPLE from sage and lemon verbena is responsible for the maintenance of intestinal health, supporting gut homeostasis and increasing the integrity of the intestinal epithelium, which suggests that this phytogenic may be considered as a promising sustainable functional additive for aquafeeds.

## Introduction

Aquaculture will supply the majority of aquatic dietary protein by 2050 (1), playing a relevant role in food security and supply, and poverty alleviation (2). The sustained growth of aquaculture is highly dependent on the intensification of production (3), sustainable feed formulations (4) and generating farming conditions supporting fish health and welfare (5). Among the former concepts, disease is considered a main persistent threat to intensive fish farming, which represents an estimated US$6 billion loss per annum at a global scale (5). Under this scenario, aquaculture depends on the use of antibiotics to fight against infectious diseases that threatens production (6), with emerging infectious diseases forecast to increase with warmer temperatures (7). However, their use tend to result in the emergence of antimicrobial resistant bacteria, which may not represent a direct threat in terms of aquatic food consumption, but they could directly impact production itself by lowering drug efficacy, decreasing the animal’s immune system and selecting more virulent strains of pathogens (7). Considering the above-mentioned reasons, along with the increasing public awareness regarding food safety issues and the environmental impact linked to antibiotics’ use and animal welfare (7, 8), the development of functional feeds focused on promoting and modulating the host’s immune response has been encouraged during the last decade (9–12).

Functional feeds are recognized for promoting the growth, welfare and health of farmed animals coupled with an improvement and/or modulation of their immune system, as well as inducing physiological benefits beyond traditional feeding practices (13). In this sense, by preventive health management through the diet, fish can divert more energy to somatic growth and reduce biological energy reserves needed to fight disease or stress resistance (14). Furthermore, they can be used in addition to chemotherapeutic agents and vaccines (15). Among the long list of feed additives used in animal production (9, 16), phytogenics derived from herbs, spices, medicinal or aromatic plants are residue-free, unlike synthetic antibiotics, and are safe ingredients for sustainable feeds (11, 17, 18). Although the mode of action of most phytogenic feed additives has not yet been fully elucidated, they are well-known for their antimicrobial, immunomodulatory, antioxidative, and growth-promoting effects in livestock (17) and aquatic animals (9, 18).

In a recent study from our research group, we showed that a phytogenic feed additive from sage (*Salvia officinalis*, Lamiaceae) and lemon verbena (*Lippia citriodora*, Verbenaceae) is an effective additive for aquafeeds since its inclusion at 0.1% in diets with low fishmeal (FM) content not only improved some key performance indicators (i.e., growth and feed efficiency performances), but also promoted fish systemic immunity. In particular, an *ex vivo* study with splenocytes from fish fed this phytogenic exposed to lipopolysaccharide (LPS) showed an up-regulation of genes involved in non-specific immune response, as well as pro- and anti-inflammatory cytokines, surface T-cell marker cd4, and antioxidative stress enzymes (14). However, the effects of this phytogenic feed additive still needs to be explored in terms of local immune response, especially at the intestinal level, since optimal health and functionality of the intestinal mucosa is essential for sustainable animal production (19, 20). This is of special relevance under the current scenario in which aquafeeds are formulated with low levels of fishmeal (FM) (21), since several studies have indicated that low FM diets compromised systemic immunity (22–25), as well as gut immune response (26, 27). In this context, intestinal immunity is a key factor in maintaining the general health of aquatic animals (20). The intestinal mucosa is a complex organ composed of the digestive epithelium with its specific structure, the gut-associated lymphoid tissue (GALT), and the mucus overlying the epithelium with its commensal microbiota (19). Furthermore, the intestinal epithelium acts as a selectively permeable barrier for dietary nutrients, electrolytes and water, while maintaining an effective defense against pathogens and tolerance towards dietary antigens. Thus, the GALT is reputed for mediating mucosal innate and adaptive immune responses, as well as being a key element for proper distinction between pathogens and commensal microbiota inhabiting the intestine (28–30).

Considering the importance of interaction between the diet and the gut, in the current study we evaluated the transcriptomic profile and histochemical properties of mucins stored in goblet cells of the intestine in juvenile gilthead seabream (*Sparus aurata*) fed a functional diet containing a medicinal plant leaf extract (MPLE) from sage and lemon verbena. This species is recognized as the most important Mediterranean aquaculture fish species in terms of volume and economic value (31). For this purpose, we focused on the modulation of the intestinal mucosa functionality and health by the above-mentioned phytogenics when included in a diet with low fishmeal levels.

## Material and Methods

### Diets, Fish and Rearing Conditions

Two isoproteic (48% crude protein), isolipidic (17% crude fat) and isoenergetic (21.7 MJ kg^-1^) experimental diets were formulated with a low FM content (7% FM) as described in Salomón et al. (14). Diets, named as control and MPLE, only differed in their content of the feed additive evaluated, the MPLE obtained from sage and verbena leaves, which was included in the MPLE diet at 0.1% (**Table 1**). The MPLE was obtained by NATAC Biotech SL using water/ethanol extraction (plant leaf extract ratio 5:1) and characterized as described in Arthur et al. (32) and Wójciak-Kosior et al. (33). The tested extract (proximate composition: 73% carbohydrates, 2% crude lipids, <1% crude proteins, 5% salts and 4% water) contained 10%, ursolic acid, 3% other triterpenic compounds, 2% verbascoside and <1% polyphenols. Thus, the content in plant-derived bioactive compounds in the MPLE diet was 100 ppm ursolic acid, 30 ppm other triterpenic compounds, 60 ppm verbascoside and <10 ppm polyphenols. This phytogenic was incorporated in the mixture prior to extrusion. In brief, all powder ingredients were mixed in a double-paddle mixer (model RM90L, Mainca, Spain) and ground (below 250 µm) in a micropulverizer hammer mill (model SH1, Hosokawa-Alpine, Germany). Diets were manufactured with a twin-screw extruder (model BC45, Clextral, France) with a screw diameter of 55.5 mm. Extruded pellets were dried in a vibrating fluid bed dryer (model DR100, TGC Extrusion, France). Oils were added by vacuum coating (model PG-10VCLAB, Dinnissen, The Netherlands). Immediately after coating, diets were packed in sealed plastic buckets and shipped by road to the facilities at IRTA Sant Carles de la Ràpita, Spain. Both extruded diets (pellet size: 2 mm) used in this trial were manufactured by SPAROS Lda. (Portugal) and kept at 4°C until their administration. Proximate composition of the extract and experimental diets have been described in Salomón et al. (14).

**Table 1:** List of ingredients and proximal composition of experimental diets; control and a basal diet supplemented with a medicinal plant leaf extract (MPLE) obtained from sage (*Salvia officinalis*) and lemon verbena (*Lippia citriodora*).

Ingredients, %	Control diet	MPLE diet
Fishmeal LT70	7.0	7.0
Soy protein concentrate	21.0	21.0
Pea protein concentrate	12.0	12.0
Wheat gluten	12.0	12.0
Corn gluten	12.0	12.0
Soybean meal 48	5.0	5.0
Wheat meal	10.4	10.4
Fish oil (SAVINOR)	15.0	15.0
Vitamin and mineral Premix PV01	1.0	1.0
Soy lecithin – Powder	1.0	1.0
Binder (guar gum)	1.0	1.0
MCP	2.0	2.0
L-Lysine	0.3	0.3
L-Tryptophan	0.1	0.1
DL-Methionine	0.2	0.2
MPLE	–	0.1
**Proximate composition**		
Crude protein, %	48.37	48.37
Crude fat, %	17.19	17.21
Fiber, %	1.52	1.52
Ash, %	5.88	5.88
Gross Energy, MJ/kg	21.62	21.62

MPLE, medicinal plant leaf extract obtained from sage (Salvia officinalis) and lemon verbena (Lippia citriodora). Proximal compositions of the diets were according to Salomon et al. (14), following the AOAC guidelines.

A total of 300 gilthead seabream (body weight, BW = 5.0 ± 0.2 g; mean ± standard deviation) were obtained from a commercial fish farm, Piscicultura Marina Mediterránea SL (Andromeda Group, Burriana, Spain) and transported to the experimental facilities of IRTA in Sant Carles de la Ràpita (Tarragona, Spain). Then, fish were acclimatized for three weeks in 450 L tanks connected to a water recirculation system (IRTAmar™) at an initial density of 2 kg m^-^³. Acclimation was conducted in the same experimental tanks (450 L) where the nutritional experiment was carried out. Just before the start of the trial, all animals were gently anesthetized (50 mg l^-1^ MS-222, Sigma-Aldrich, Madrid, Spain), individually measured in BW (26.0 ± 0.2 g) and distributed homogeneously among the eight experimental tanks (n = 35 fish per tank; 4 replicate tanks per experimental diet). During the trial that lasted 92 days, fish were fed at the daily rate of 3.0% of the stocked biomass, which approached apparent satiation as described in Salomón et al. (14). Feed ration was regularly adjusted by means of intermediate samplings along the trial. At the end of the trial, all fish were anesthetized as previously indicated and measured for individual BW. Fish performance in terms of survival (S _control diet_ = 98.0 ± 1.0%; S _MPLE diet_ = 99.0 ± 0.8%), growth (BW _control diet_ = 173.8 ± 8.2 g; BW _MPLE diet_ = 189.6 ± 5.0g) and feed conversion ratio (FCR _control diet_ = 1.25 ± 0.04; FCR _MPLE diet_ = 1.10 ± 0.04) was already published in Salomón et al. (14). Twelve fish from each experimental diet (n total= 3 fish per tank replicate) were randomly selected and sacrificed with an overdose of anesthetic (300 mg l^-1^ MS-222) for gut transcriptomic and histological analyses. Sacrificed animals were eviscerated and mid-anterior intestine samples (1-1.5 cm length per specimen) were put in RNAlater™ (Invitrogen, Thermo Fisher Scientific, Lithuania), incubated overnight at 4°C, and stored at -80°C until RNA extraction. This region of the intestine (mid-anterior section) was chosen due to its specialized immunological functionality when compared with other intestinal sections (34). In addition, a similar piece of tissue was also dissected and fixed in 10% v/v neutral formaldehyde (pH: 7.2 ± 0.01) buffered with sodium phosphate (0.1M) for histological and histochemical purposes.

Water temperature (25.1 ± 1.5°C, range: 22-27°C), oxygen (6.1 ± 0.2 mg l^-1^) (OXI330, Crison Instruments), and pH (7.5 ± 0.01) (pHmeter 507, Crison Instruments, Barcelona, Spain) were daily controlled. Salinity (35‰) (MASTER-20 T; ATAGO Co. Ltd), ammonia (0.13 ± 0.1 mg NH_4_^+^ l^-1^) and nitrite (0.18 ± 0.1 mg NO^-^ l^-1^) levels (HACH DR9000 Colorimeter, Hach^®^, Spain) were weekly monitored. The trial was run under natural photoperiod according to the season of the year (August to November; 40°37’41” N).

### Transcriptional Analysis

#### RNA Isolation and Quality Control

Total RNA was extracted from mid-anterior intestine of individual fish (n = 12 fish per dietary treatment) using the RNeasy^®^ Mini Kit (Qiagen, Germany). Total RNA from each individual sample was eluted in a final volume of 35 μL nuclease-free water and treated with DNAse using the DNA-free™ DNA Removal Kit according to manufacturer’s instructions (Invitrogen, Thermo Fisher Scientific, Lithuania). Total RNA concentration and purity were quantified using a Nanodrop-2000^®^ spectrophotometer (Thermo Scientific, USA) and stored at -80°C until analysis. Prior to hybridization with microarrays (3 pooled RNA per dietary condition), four individual RNA samples were pooled by mixing a volume of 1.5 μL [(RNA) = 133.33 ng/µL] per individual sample [final volume = 6 µL; (RNA) = 133.33 ng/µL] and checked for integrity using an Agilent 2100 Bioanalyzer (Agilent Technologies, Spain). Pooled RNA analyzed in this study were selected by the criteria of a RIN value > 8.5 (**Supplementary Figure 1**). This methodological approach of pooling RNA from four different specimens in each replicate (n = 3 microarray replicates) allowed authors evaluating population’s variability (n = 4 replicate tanks; 1 fish per tank in each pooled RNA; N = 12 animals); however, the information regarding individual variability was lost with this choice.

#### Microarray Hybridization and Analysis

Transcriptional analysis was carried out using the Aquagenomics *Sparus aurata* oligonucleotide microarray v2.0 (4 x 44 K) (SAQ) platform. The detailed information about the platform and the transcriptomic raw data for all samples included in this current analysis is available through the public repository Gene Expression Omnibus (GEO) at the US National Centre for Biotechnology Information (NCBI) (accession number GPL13442 and GSE166558, respectively).

Transcriptomic analysis from both experimental groups were conducted as described by Reyes-López et al. (35). Briefly, 200 ng of total RNA from each sample pool was reverse transcribed along with Agilent One-Color RNA spike-in kit (Agilent Technologies, USA). Then, total RNA was used as template for Cyanine-3 (Cy3) labelled cRNA synthesis and amplification with the Quick Amp Labelling kit (Agilent Technologies). cRNA samples were purified using the RNeasy micro kit (Qiagen). Dye incorporation and cRNA yield were checked with the NanoDrop ND-2000^®^ spectrophotometer. Then, 1.5 mg of Cy3-labeled cRNA with specific activity > 6.0 pmol Cy3/mg cRNA were fragmented at 60°C for 30 min, and then the samples were mixed with hybridization buffer and hybridized to the array (ID 025603, Agilent Technologies) at 65°C for 17 h using the Gene expression hybridization kit (Agilent Technologies). The microarray washes were conducted as recommended by the manufacturer using Gene expression wash buffers (Agilent Technologies) and stabilization and drying solution (Agilent Technologies). Microarrays slides were scanned with an Agilent Technologies Scanner (model G2505B); spot intensities and other quality control features were extracted with Agilent’s Feature Extraction software version 10.4.0.0 (Agilent Technologies). Quality reports were checked for each array. Although validation by mean of qPCR is required when there is a high risk of obtaining paralog genes and unspecific hybridization; in our study, we used an oligonucleotide-based microarray, which has probes with a reduced number of bases and high affinity, which avoids the necessity of conducting the validation of gene expression results by qPCR validation.

#### Transcripteractome

The Search Tool for the Retrieval of Interacting Genes (STRING) public repository version 11.0 (https://string-db.org) was used in order to obtain the gut transcripteractome for those differentially expressed genes (DEGs) from fish fed the MPLE diet in comparison to the control group (*P*- value < 0.05) (36). This functional network analysis has gained increasing attention because of the association between different genes sorted in different clusters that may have complementary functions into a biological context of response (35). A protein-protein interaction (PPI) network of DEGs was conducted with a high-confidence interaction score (0.9) using *Homo sapiens* as model organism. Furthermore, to confirm matches of the genes acronyms tag between both *H. sapiens* and gilthead seabream species Genecards (37) and Uniprot (38) databases were used. In order to confirm match of gene acronyms between both *H. sapiens* and gilthead seabream species, human orthology identification based on gene/protein name was accessed through the Genecards (www.genecards.org) (37) and Uniprot (www.uniprot.org) (UniProt, 2019) databases. Additionally, protein-protein BLAST (BLASTp) were analyzed (E-value < 10^-7^; query cover > 95%). Gene ontology (GO) enrichment analysis for the DEGs were also performed by STRING (*P* < 0.05).

### Histological and Histochemical Analyses

Samples (n = 12 fish per experimental diet) were embedded in paraffin and sagitally sectioned (5-6 µm). A total of 576 sections (2 per each sample x 24 samples x 12 techniques) were used for histological and histochemical purposes. Two sections per each sample were stained with hematoxylin–VOF for descriptive purposes; the rest were used for evaluating the histochemical properties of epithelial and mucous cells. In brief, Schiff, Periodic Acid Schiff (PAS), diastase-PAS and Alcian Blue (AB) pH 2.5, 1 and 0.5 (carboxylated and sulphated glycoconjugates/glycoproteins) techniques were used for studying carbohydrate distribution. Furthermore, several horseradish peroxidase (HRP) conjugated lectins (Sigma-Aldrich, Spain) were used for proper characterization of different glucidic residues bound to the glycoconjugates; in particular, *Canavalia ensiformes*/ConA (Mannose and/or Glucose), *Ulex europeus*/UEA-I (L-Fucose), *Triticum vulgaris*/WGA (N-acetyl-D-glucosamine and/or N-acetylneuraminic acid, NeuNAc/sialic acid/NANA), Glycine max/SBA (α-N-acetyl-D-galactosamine) and *Sambucus nigra*/SNA (NeuNAc/sialic acid/NANA). Lectin concentrations ranged between 15 µg ml^-1^ to 30 µg ml^-1^. Regarding negative controls, omission of the respective lectin, substitution of lectin-HPR conjugates by TBS and treatments with different enzymes were performed according to Sarasquete et al. (39). The peroxidase activity was visualized with 3,3-diaminobenzidine tetra hydrochloride/DAB and hydrogen peroxide (0.05%). All the techniques were performed according to Pearse (40) and following proper standardized techniques and protocols (41). All reagents were purchased from Sigma Chemical Co. St Louis, MO, USA.

Histological images were taken with a Leitz Wetzlar microscope with a built-in SPOT Insight Color camera (Ernst Leitz Wetzlar GmbH, Germany). Results were manually registered using a semi-quantitative assessment scoring based on color intensity scores (0, negative; 1, weak; 2, moderate; 3, intense; 4, very intense) from four independent observers, comparing the sections of the control with the experimental diet. The mucous cell count was determined in four different sites of each histological section, and the number of cells expressed per length unit of the basal lamina of the mucosal epithelium (1 mm) according to Yamamoto et al. (42).

### Ethics Statement

All animal experimental procedures were complied with the Guiding Principles for Biomedical Research Involving Animals (EU2010/63), the guidelines of the Spanish laws (law 32/2007 and RD 1201/2015), and authorized by the Ethical Committee of the Institute for Research and Technology in Food and Agriculture (IRTA, Spain) for the use of laboratory animals.

### Statistics

Extracted raw data from microarrays were imported and analyzed with Genespring version 14.5 GX software (Agilent Technologies). The 75% percentile normalization was used to standardize arrays for comparisons and data were filtered by expression. An unpaired t-test was conducted without correction to identify those DEGs between fish fed control and MPLE diets. The Principal Component Analysis (PCA) was carried out using GeneSpring software, four eigenvectors were calculated to describe the aggrupation of the MPLE and control groups in a 3D plot. Venn diagram and the hierarchical heatmap were all obtained also with Genespring (version 14.5 GX software, Agilent). Changes in the number of mucous cells between experimental diets were analyzed by means of an unpaired t-test assuming data homoscedasticity (Barlett’s test). All the analysis were performed using GraphPad PRISM 7.00. The level of significance was set at *P* < 0.05 for all statistical tests.

## Results

### Organization of the Intestine and Mucins’ Histochemistry Produced by Goblet Cells

**Figure 1:**
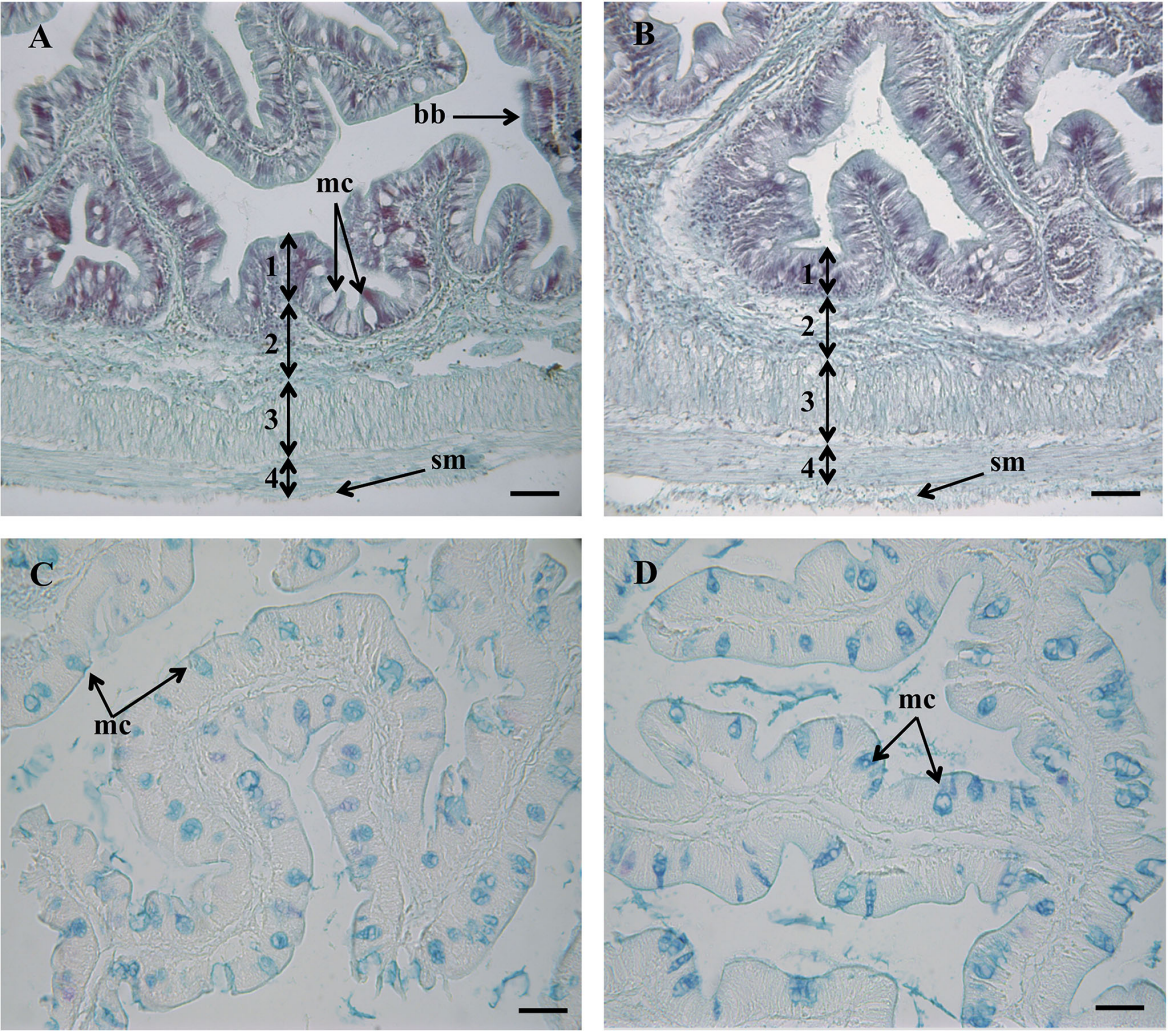
Histological organization in the intestine of gilthead seabream (*Sparus aurata*) fed a control diet **(A)**, and medicinal plant leaf extract (MPLE)-supplemented diet **(B)**. Numbers indicate the different intestinal layers: (1) mucosa; (2) lamina propria-submucosa; (3) circular muscle layer; (4) longitudinal muscle layer. bb: brush border; mc: mucous cells; sm: serous membrane. Histochemical properties of mucins secreted by intestinal goblet cells with regard to their content on carboxylated and/or sulphated acidic groups (Alcian Blue pH = 2.5) from fish the control diet **(C)** and the MPLE-supplemented diet **(D)**. mc, mucous cells. The MPLE included in the basal diet was obtained from sage (*Salvia officinalis*) and lemon verbena (*Lippia citriodora*). Staining: hematoxylin-VOF **(A, B)**, Alcian Blue pH = 2.5/PAS **(C, D)**. Scale bar = 50 µm.

**Figure 2:**
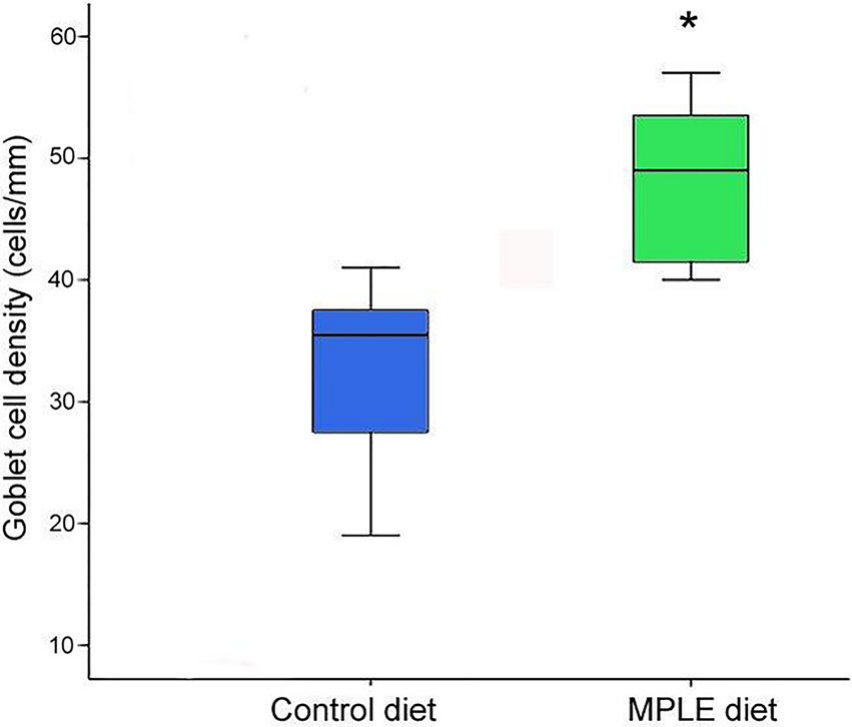
Goblet cell density in the intestine of gilthead seabream (*Sparus aurata*) fed a control or a medicinal plant leaf extract (MPLE)-supplemented diet. The MPLE included in the basal diet was obtained from sage (*Salvia officinalis*) and lemon verbena (*Lippia citriodora*). The asterisk indicates statistically significant differences among dietary treatments (t-test; *P* < 0.05).

In both experimental groups, the intestinal mucosa was lined by a simple columnar epithelium with basal nuclei, basophilic cytoplasm and prominent brush border with scattered goblet cells. The organization of the lamina propria-submucosa and muscular layers was normal. No signals of histological alterations associated to inflammatory processes in the intestine were observed in fish fed the MPLE diet compared to the control group (**Figures 1A, B**). Fish fed the MPLE diet showed a higher density of goblet cells along the intestinal epithelium compared to fish fed the control diet (**Figure 2**).

**Figure 3:**
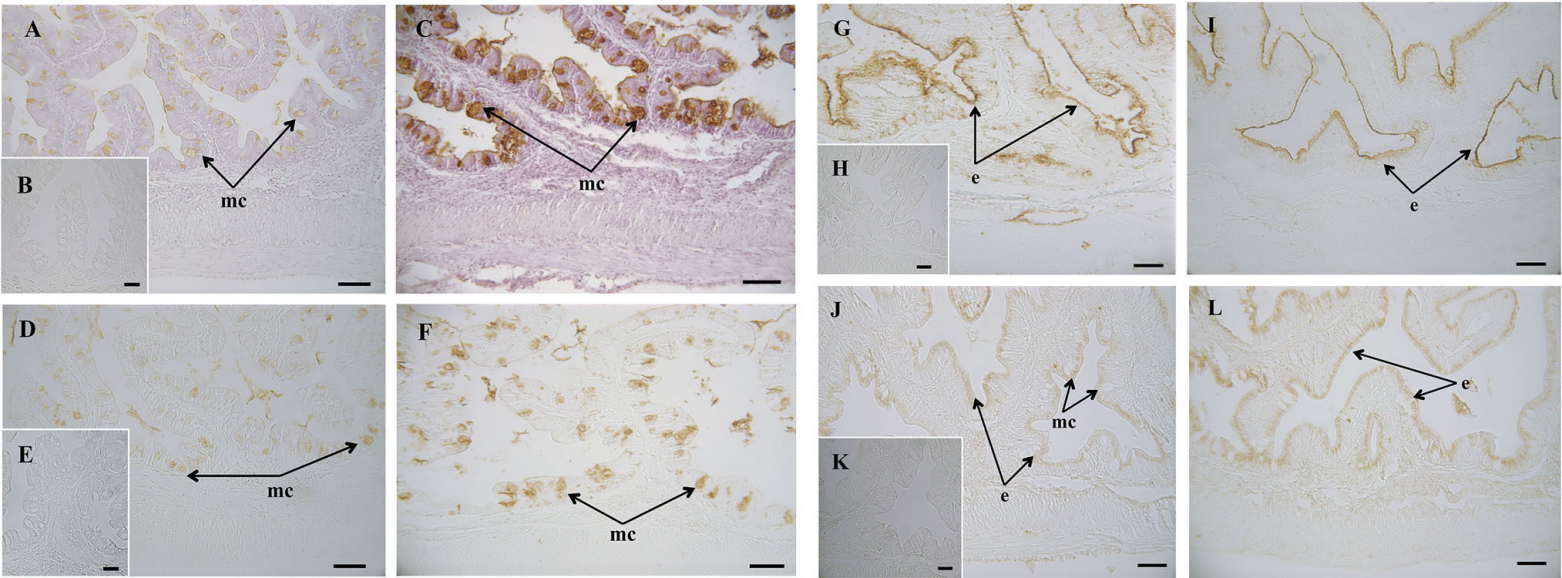
Histochemical localization of glycoconjugates containing sugar residues in the intestine of gilthead seabream (*Sparus aurata*) fed a control or a medicinal plant leaf extract (MPLE)-supplemented diet. Presence of glycoconjugates containing N-acetyl-D-glucosamine and/or N-acetylneuraminic acid residues in mucous cells of *S. aurata* fed a control **(A)** (**B**: negative control) or a MPLE-supplemented diet **(C)**. Note the increase in affinity for WGA lectin in the mucinous content of goblet cells from MPLE diet. Glycoconjugates containing α-N-acetyl-D-galactosamine residues in mucous cells of *S. aurata* fed a control **(D)** (**E**: negative control) or a MPLE-supplemented diet **(F)**. Results denote a moderate increase in affinity for the SBA lectin in the mucous cells from MPLE diet. Histochemical detection of glycoconjugates containing N-acetylneuraminic acid/sialic acid residues in the intestine from control **(G)** (**H**: negative control) or MPLE group **(I)**. Note the decrease in affinity for the SNA lectin in the intestinal epithelium of *S. aurata* fed a MPLE-supplemented diet. Glycoconjugates containing α-mannose/α-glucose residues in intestine from *S. aurata* fed a control **(J)** (**K**: negative control) or a MPLE-supplemented diet **(L)**. Observe the increase in affinity for the ConA lectin in the intestinal epithelium of *S. aurata* fed a MPLE-supplemented diet. Mucous cells were negative for ConA lectin in both control and MPLE groups. e: epithelium; mc: mucous cells. The MPLE included in the basal diet was obtained from sage (*Salvia officinalis*) and lemon verbena (*Lippia citriodora*). Scale bar = 50 µm.

Regarding the histochemical properties of mucins in goblet cells from the anterior intestine, results showed a variable richness of neutral glycoproteins (PAS and diastase-PAS positive) (**Table 2**). In addition, mucins from goblet cells showed a mixture of carboxylated (AB pH = 2.5) and sulphated acidic groups (weak and strongly ionized; AB pH = 1.0 and pH 0.5, respectively) (**Table 2** and **Figure 1C**). Furthermore, a specific affinity for WGA, SNA and SBA lectins was detected in the mucinous content of goblet cells (**Table 2** and **Figures 3A–L**). Moreover, no variations were detected in the distribution of mucosal cell glycoconjugates between the upper and the bottom areas of the intestinal folds. When comparing both dietary groups, the dietary administration of the MPLE modified the composition of glycoproteins of mucins produced by goblet cells, with an increase in the staining intensity of neutral mucins, as well as in mucins rich in carboxylated and weakly sulphated glycoconjugates (**Table 2** and **Figures 1C, D**). In addition, an increase in affinity for WGA and SBA lectins and a decrease in the affinity for the SNA lectin was found in the mucinous content of goblet cells, whereas no changes were detected regarding ConA and UEA-I lectins (**Table 2** and **Figures 3A–L**).

**Table 2:** Histochemical characteristics and lectin affinity of mucins produced by goblet cells from anterior intestine of gilthead seabream (*Sparus aurata*) fed the control and this basal diet supplemented with a medicinal plant leaf extract (MPLE) obtained from sage (*Salvia officinalis*) and lemon verbena (*Lippia citriodora*).

	Control diet	MPLE diet
**General histochemistry**		
Neutral glycoproteins	1 - 2	2 - 3
Carboxylated glycoproteins	1 - 3	3
Weakly inonised sulphated glycoconjugates	2 - 3	3
Strongly ionised sulphated glycoconjugates	2 - 3	2 - 3
**Lectin histochemistry**		
ConA (Man/Glu)	0	0
WGA (βGlcNAc>>NeuNAc/sialic acids/NANA)	2 - 3	3
SNA (Neur5Acα2; sialic acids/NANA)	1 - 3	0
SBA (α/β GalNAc)	0 - 3	1 - 3
UEA-I (Fuc)	0	0

Semi-quantitative assessment scoring based on color intensity scores: 0, negative (non detected); 1, weak; 2, moderate; 3, intense; 4, very intense.

### Microarrays and Gut Transcripteractome

**Figure 4:**
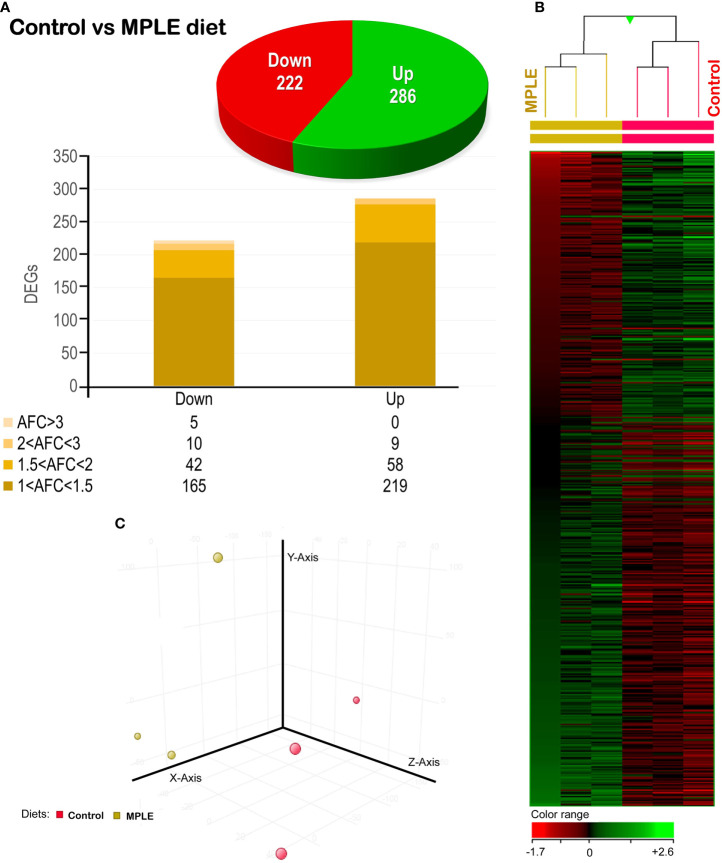
Differential expression analysis of the gilthead seabream (*Sparus aurata*) mid-anterior intestine transcriptomic response to MPLE diet. **(A)** Distribution of the differential expressed genes (DEGs) obtained from the microarray-based transcriptomic analysis. The absolute fold change (AFC) indicates the fold-change magnitude interval of response. **(B)** Hierarchical clustering of the gilthead seabream mid-anterior intestine transcriptomic response for the control and MPLE diet, based in similitude patterns of the differentially expressed genes (DEGs) detected from three sample pools per dietary group. Data of the six microarrays are depicted, one for each representing the pooled RNA. Genes from each replicate are ordered from lower to higher AFC intensities using the left sample (randomly chosen) as a reference for ordering the other five samples (GeneSpring version 14.5 GX software; Agilent Technologies). Both increased and decreased gene expression pattern is shown in green and red, respectively, according to the color range (bottom). All transcripts represented are statistically significant (*P* < 0.05). **(C)** Principal component analysis (PCA) of the DEGs for the gilthead seabream intestine in response to the control (yellow node) and MPLE-supplemented diet (red node). The MPLE included in the basal diet was obtained from sage (*Salvia officinalis*) and lemon verbena (*Lippia citriodora*).

A total number of 506 DEGs were found when comparing the transcriptomic profiling of the intestine from gilthead seabream fed the control and MPLE diets (*P* < 0.05; **Figure 4A** and **Supplementary Table 1**). Common segregation among the pool samples within the same dietary treatment was observed in the hierarchical clustering for the gut transcriptomic response based in similitude patterns of the DEGs response (*P* < 0.05; **Figure 4B**). The observed segregation among dietary treatments is supported by the PCA analysis for the analyzed samples (**Figure 4C**); in particular, four eigenvectors were calculated and three principal components were plotted, explaining the 83.2% of the total variability [component 1 (X-axis): 45.5%; component 2 (y-axis): 24.4%; component 3 (z-axis): 13.3%]. The detailed analysis of gene absolute fold-change (AFC) revealed that genes were mostly up-regulated in fish fed the MPLE diet (56.3% of DEGs), while its modulation was moderate in terms of AFC intensity (**Figure 4**). In particular, 285 of the above-mentioned DEGs were up-regulated with 219 of them within the 1.0 ≤ AFC ≤ 1.5 interval, 58 DEGs were grouped within the 1.5 ≤ AFC ≤ 2.0, and the last 9 DEGs up-regulated were grouped 2.0 ≤ AFC ≤ 3.0. In contrast, 221 genes were significantly down-regulated and grouped in the range -1.0 ≤ AFC ≤ -3.0. (*P* < 0.05). In particular, 164 DEGs were mainly concentrated in the -1.0 ≤ AFC ≤ -1.5 range. Among them, 42 DEGs were grouped within the -1.5 ≤ AFC ≤ -2.0 interval, 10 DEGs were felt within the -2.0 ≤ AFC ≤ -3.0 category, while only 5 were found to have an AFC higher than -3 (**Figure 4**).

From the whole set of DEGs, a functional network analysis was performed. The transcripteractome showed 244 genes with 552 interactions (edges). The remaining 264 DEGs were classified as unknown genes; thus, they were excluded from the analysis. According to GO results and their respective annotation hierarchy, three main representative groups of genes were identified in the transcripteractome among the totality of biological processes obtained from the enrichment analysis (**Supplementary Table 2**): (1) immune system processes, (2) cellular development and organization, and (3) cellular catabolism (**Supplementary Tables 3–5**). **Table 3** summarizes the most relevant DEGs in fish fed the MPLE diet in relation to the above-mentioned biological processes.

**Table 3:** List of the most relevant DEGs related to three main representative biological processes identified by the transcripteractome (1, immune system processes; 2, cellular development and organization; 3, cellular catabolism) in fish fed the MPLE diet.

Gene description	Gene acronym	Biological processes	AFC	*P*-value
CYLD lysine 63 deubiquitinase	*cyld*	1	2,374	0,027
Actinin Alpha 4	*actn4*	2	1,631	0,022
Proteasome 26S subunit, non-ATPase 1	*psmd1*	1, 3	1,582	0,015
Ubiquitin like modifier activating enzyme 1	*uba1*	1, 3	1,570	0,020
Chitinase 3 Like 1	*chi3l1*	1	1,549	0,046
Proteasome 26S subunit, non-ATPase 5	*psmd5*	1, 3	1,537	0,041
Erythrocyte membrane protein band 4.1	*epb41*	2	1,476	0,032
Caspase 8	*casp8*	1	1,472	0,004
Sequestosome 1	*sqstm1*	1, 3	1,413	0,031
Proteasome subunit beta 5	*psmb5*	1, 3	1,408	0,020
Zinc and ring finger 3	*znrf3*	3	1,336	0,038
RAP1B, member of RAS oncogene family	*rap1b*	2, 3	1,321	0,004
LIM domain binding 1	*ldb1*	2, 3	1,311	0,019
COP9 signalosome subunit 4	*cops4*	1, 3	1,255	0,001
Ubiquitin specific peptidase 4	*usp4*	1, 3	1,231	0,037
Ras homolog family member A	*rhoa*	2	1,222	0,047
Fibroblast growth factor 18	*fgf18*	3	1,206	0,041
Mechanistic target of rapamycin kinase	*mtor*	1, 3	1,176	0,021
Proteasome subunit beta 2	*psmb2*	1, 3	1,167	0,023
Phosphoglycerate kinase 1	*pgk1*	3	1,164	0,046
Ubiquitin conjugating enzyme E2 A	*ube2a*	1, 3	1,102	0,039
Ubiquitin conjugating enzyme E2 G1	*ube2g1*	1, 3	-1,100	0,001
C-Type lectin domain family 4 member e	*clec4e*	1	-1,154	0,013
NLR family card domain containing 3	*nlrc3*	1	-1,315	0,020
Signal transducer and activator of transcription 3	*stat3*	1, 3	-1,367	0,0005
BCL2, apoptosis regulator	*bcl2*	1	-1,378	0,038
Ubiquitin specific peptidase 7	*ups7*	1, 3	-1,385	0,044
Galectin 1	*lgals1*	1, 3	-1,452	0,020
Ubiquitin conjugating enzyme E2 D2	*ube2d2*	1, 3	-1,457	0,021

**Figure 5:**
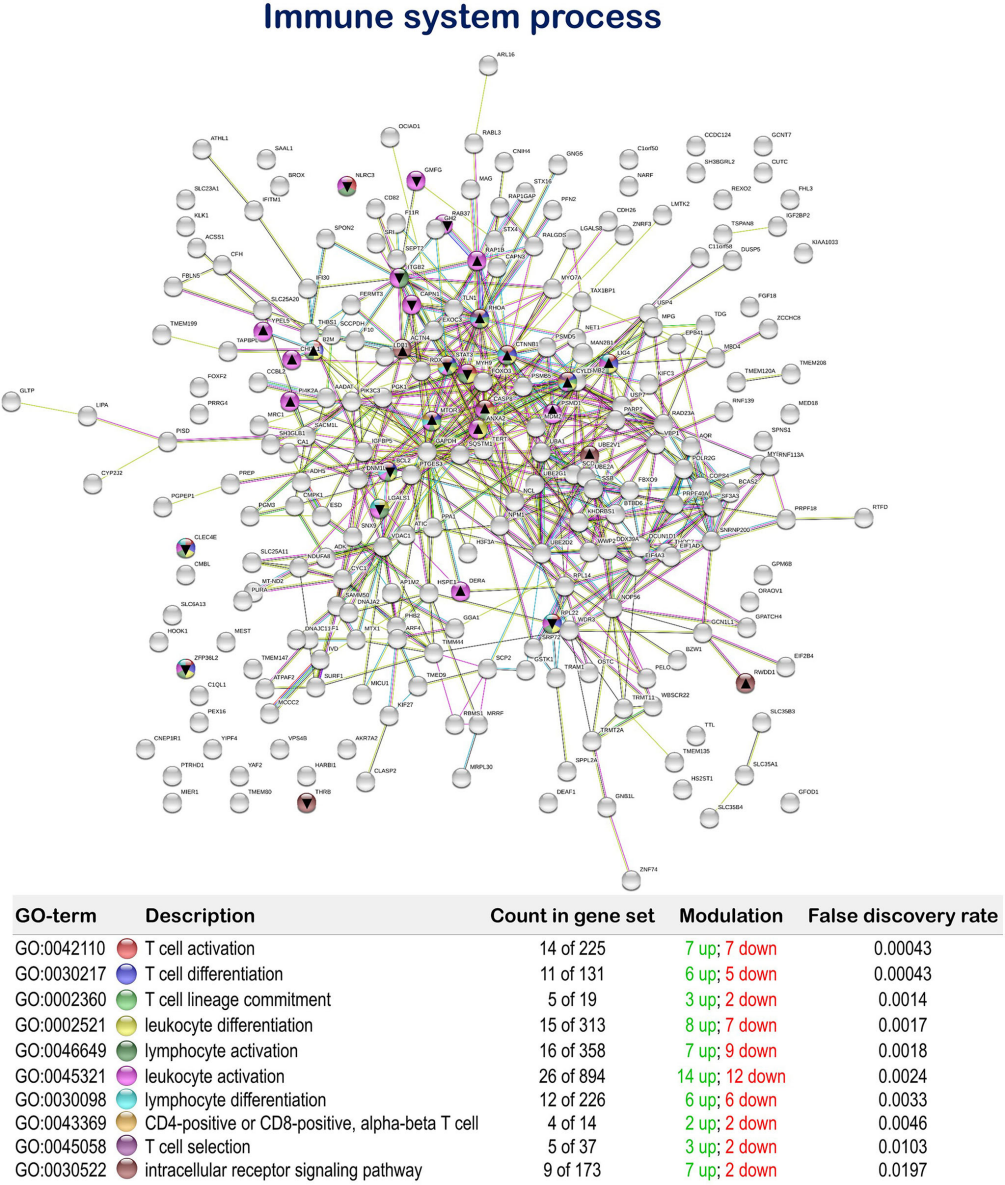
Immune system process related Protein-Protein Interactions Network (PPI) network of the differentially expressed genes (DEGs) in the mid-anterior intestine of juvenile gilthead seabream (*Sparus aurata*) fed the mixture of MPLE obtained from sage (*S. officinalis*) and lemon verbena (*L. citriodora*) (see also **Supplementary Table 3**). Nodes colors indicate the biological processes for each DEG represented. Gene Ontology (GO) definitions, count of DEGs within each biological processes and respective false discovery rate are described in the graphical figure legend. ▲ nodes represent up-regulated genes and ▼ nodes represent down-regulated genes. Graphic keys and network stats are indicated in the graphical figure legend. Network Stats: number of nodes: 243; number of edges: 550; average node degree: 4.53; avg. local clustering coefficient: 0.416; expected number of edges: 423; PPI enrichment p-value: 1.98e-09. The MPLE included in the basal diet was obtained from sage (*Salvia officinalis*) and lemon verbena (*Lippia citriodora*).

Regarding the dietary regulation of biological processes related to gut immunity, 18 genes were up-regulated and 14 genes down-regulated in fish fed the MPLE diet. In particular, several relevant GOs related to immunity were obtained such as “T cell activation” (GO:0042110; 7 up-regulated genes; 7 down-regulated genes), “T cell differentiation” (GO:0030217; 6 up-regulated genes; 5 down-regulated genes), “T cell lineage commitment” (GO:0002360; 3 up-regulated genes; 2 down-regulated genes), “leukocyte differentiation” (GO:0002521; 8 up-regulated genes; 7 down-regulated genes), “lymphocyte activation” (GO:0046649; 7 up-regulated genes; 9 down-regulated genes), “leukocyte activation” (GO:0045321; 15 up-regulated genes; 12 down-regulated genes), “lymphocyte differentiation” (GO:0030098; 6 up-regulated genes; 6 down-regulated genes), “CD4+ or CD8+, α-β T cell lineage commitment” (GO:0043369; 2 up-regulated genes; 2 down-regulated genes), “T cell selection” (GO:0045058; 3 up-regulated genes; 2 down-regulated genes) and “intracellular receptor signaling pathway” (GO:0030522; 7 up-regulated genes; 2 down-regulated genes) (**Figure 5** and **Supplementary Table 3**).

**Figure 6:**
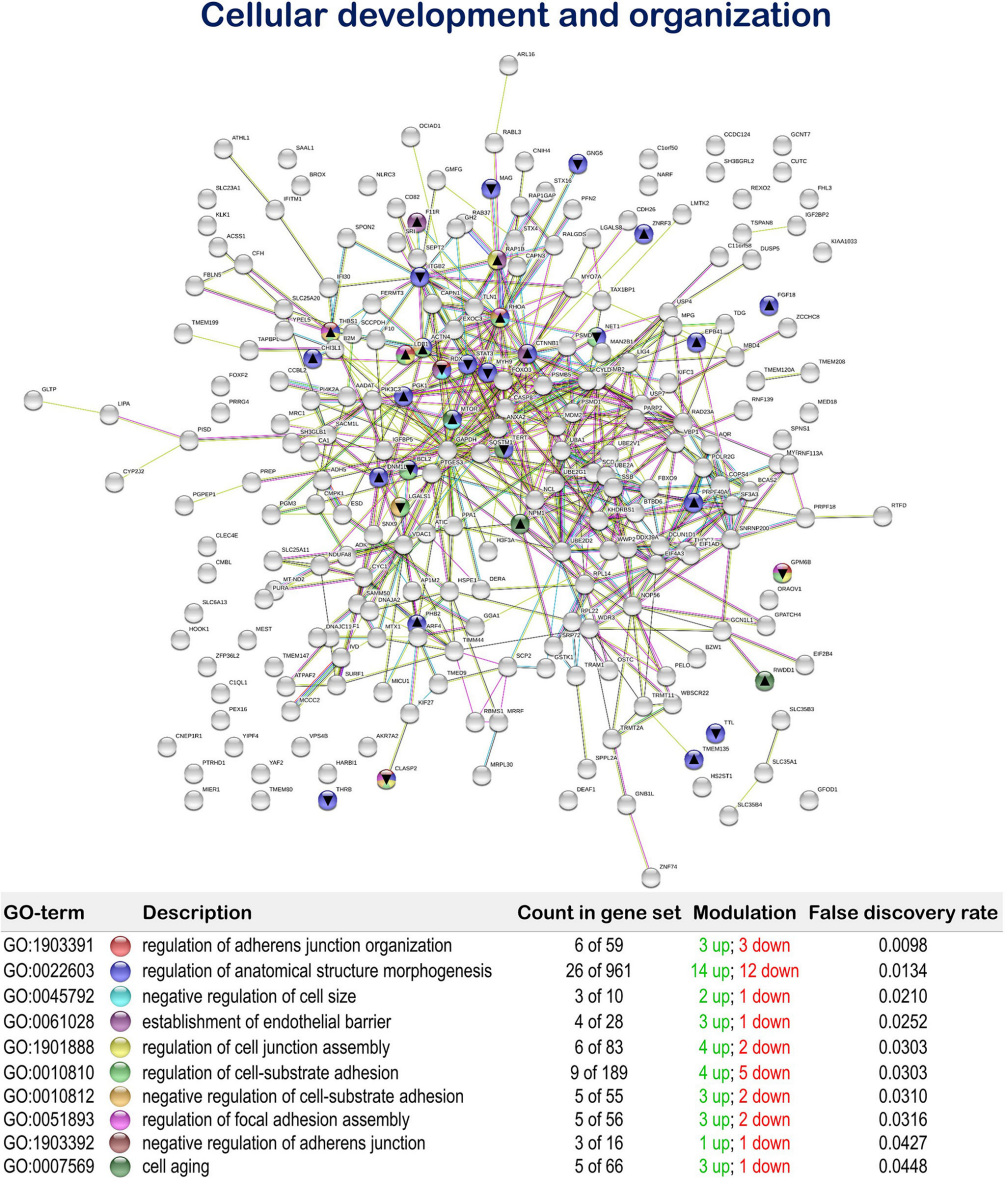
Cellular development and organization related Protein-Protein Interactions Network (PPI) network of the differentially expressed genes (DEGs) in the mid-anterior intestine of juvenile gilthead seabream (*Sparus aurata*) fed the mixture of MPLE obtained from sage (*S. officinalis*) and lemon verbena (*L. citriodora*) (see also **Supplementary Table 4**). Nodes colors indicate the biological processes for each DEG represented. Gene Ontology (GO) definitions, count of DEGs within each biological processes and respective false discovery rate are described in the graphical figure legend. ▲ nodes represent up-regulated genes and ▼ nodes represent down-regulated genes. Graphic keys and network stats are indicated in the graphical figure legend. Network Stats: number of nodes: 243; number of edges: 550; average node degree: 4.53; avg. local clustering coefficient: 0.416; expected number of edges: 423; PPI enrichment p-value: 1.98e-09. The MPLE included in the basal diet was obtained from sage (*Salvia officinalis*) and lemon verbena (*Lippia citriodora*).

Furthermore, the MPLE diet promoted the regulation of biological processes associated with cellular development and organization with 19 up-regulated and 14 down-regulated genes. Among them, we found the terms “regulation of adherens junction organization” (GO:1903391; 3 up-regulated genes; 3 down-regulated genes), “regulation of anatomical structure morphogenesis” (GO:0022603; 14 up-regulated genes; 12 down-regulated genes), “negative regulation of cell size” (GO:0045792; 2 up-regulated genes; 1 down-regulated gene), “establishment of endothelial barrier” (GO:0061028; 3 up-regulated genes; 1 down-regulated gene), “regulation of cell junction assembly” (GO:1901888; 4 up-regulated genes; 2 down-regulated genes), “regulation of cell-substrate adhesion” (GO:0010810; 4 up-regulated genes; 5 down-regulated genes), “negative regulation of cell-substrate adhesion” (GO:0010812; 3 up-regulated genes; 2 down-regulated genes), “regulation of focal adhesion assembly” (GO:0051893; 3 up-regulated genes; 2 down-regulated genes), “negative regulation of adherens junction organization” (GO:1903392; 1 up-regulated genes; 1 down-regulated genes), and “cell aging” (GO:0007569; 3 up-regulated genes; 1 down-regulated gene) (**Figure 6** and **Supplementary Table 4**).

**Figure 7:**
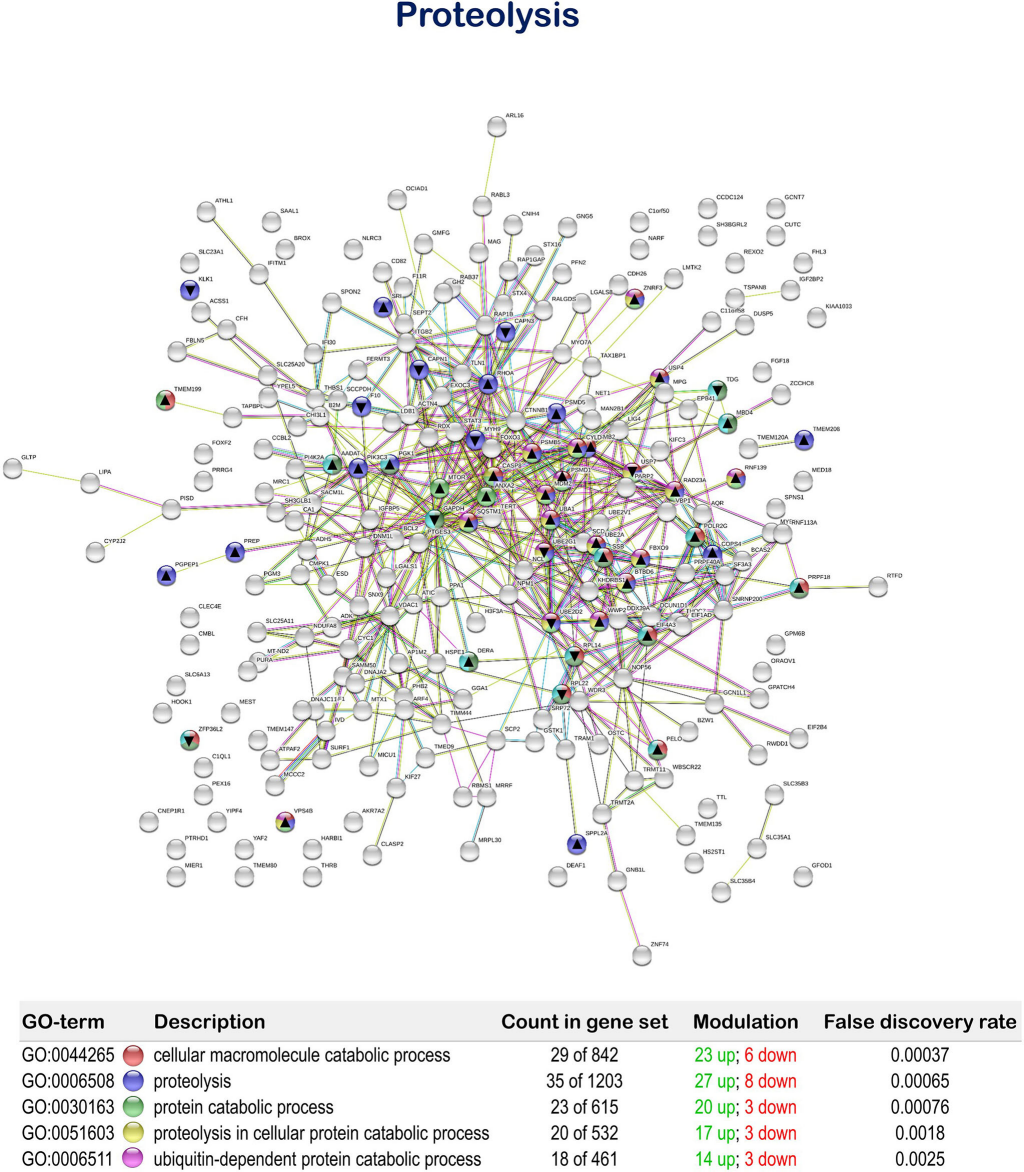
Proteolysis related Protein-Protein Interactions Network (PPI) network of the differentially expressed genes (DEGs) in the mid-anterior intestine of juvenile gilthead seabream (*Sparus aurata*) fed the mixture of MPLE obtained from sage (*S. officinalis*) and lemon verbena (*L. citriodora*) (see also **Supplementary Table 5**). Nodes colors indicate the biological processes for each DEG represented. Gene Ontology (GO) definitions, count of DEGs within each biological processes and respective false discovery rate are described in the graphical figure legend. ▲ nodes represent up-regulated genes and ▼ nodes represent down-regulated genes. Graphic keys and network stats are indicated in the graphical figure legend. Network Stats: number of nodes: 243; number of edges: 550; average node degree: 4.53; avg. local clustering coefficient: 0.416; expected number of edges: 423; PPI enrichment p-value: 1.98e-09. The MPLE included in the basal diet was obtained from sage (*Salvia officinalis*) and lemon verbena (*Lippia citriodora*).

The tested functional feed additive resulted in the positive regulation of biological processes related to cellular proteolytic processes, showing 35 up-regulated and 11 down-regulated genes. In particular, “cellular macromolecule catabolic process” (GO: 0044265; 23 up-regulated genes; 6 down-regulated genes), “proteolysis” (GO:0006508; 27 up-regulated genes; 8 down-regulated genes), “protein catabolic process” (GO:0030163; 20 up-regulated genes; 3 down-regulated genes), “proteolysis involved in cellular protein catabolic process” (GO:0051603; 17 up-regulated genes; 3 down-regulated genes) and “ubiquitin-dependent protein catabolic process” (GO:0006511; 14 up-regulated genes; 3 down-regulated genes) were obtained (**Figure 7** and **Supplementary Table 5**).

## Discussion

Considering the close relationship between diet and gut condition and the consequences on the organism and overall health, evaluating the interactions between dietary ingredients and the intestine is of special relevance due to the wide array of functions that have been associated to the gastrointestinal tract (34, 43). This is of special relevance when evaluating functional feed additives that are supposed to promote health and nutrition in farmed animals (9, 44). In this context, the present study aimed to evaluate the effects of a MPLE from sage and lemon verbena on the transcriptomic profiling, histological organization and lectin histochemistry of the intestine. Extracts from these medicinal plants are reputed for their beneficial pharmacological activities, including antiseptic, antioxidant, and anti-inflammatory properties among others (45–47). Moreover, in gilthead seabream we demonstrated that in addition to act as growth promoters, they modulated systemic immunity when splenocytes were incubated with a bacterial-type pathogen-associated molecular pattern (PAMP) like LPS (14).

### MPLE From Sage and Lemon Verbena Exert Controlled Immune and Pro-Inflammatory Responses in GALT

Fish possess innate and adaptive immune defense systems. The innate parameters are at the forefront of immune defense and are a crucial factor in disease resistance. The adaptive response of fish is essential for long-lasting immunity and is considered as a key factor in improving prophylactic strategies, and the use of functional feeds with immunomodulatory properties (20, 48). T cells are one of the main players of the adaptive immune response, being the intestine of teleosts an important site of T cells production among mucosal tissues (49, 50). In particular, T cells represent the major leucocyte population within the teleost gut (51) with relevant cytotoxic activity (52), as well as playing an important role in foreign antigen recognition and gut homeostasis (53, 54). Under the current experimental conditions, MPLE from sage and lemon verbena promoted T cell activation, differentiation and selection in gilthead seabream gut. Thus, the MPLE-supplemented diet induced up-regulation of the mammalian target of rapamycin (*mtor*) gene in the gut of gilthead seabream. The mTOR signaling pathway has been reported to promote the production of anti-inflammatory cytokines by myeloid immune cells with an important ability to limit the pro-inflammatory mediators (55) thus, playing a crucial role in intestine inflammation, epithelial morphogenesis (56, 57), as well as being an important central regulator of immune responses (58, 59). The increase in *mtor* expression may be associated to the down-regulation of *nlrc3*, which is a negative regulator of the PI3K/AKT/mTOR pathway (60), thus suggesting a balanced inflammatory dietary-induced response in the gut of fish fed the MPLE diet.

The NF-kB pathway is involved in the transcriptional regulation of several cytokines, chemokines, transcription factors, antimicrobial peptides, and interferon-stimulated genes, playing a critical role in regulating the survival, activation and differentiation of innate and adaptive immune cells (61); thus, playing a key role in regulating gut inflammation (62). In this way, several genes associated with the NF-κB pathway (*cyld*, *clec4e*, *nlrc3, chi3l1, bcl2*) were differentially regulated by the dietary supplementation of MPLE. Although scarce information is available for teleost fish, CYLD identified in rainbow trout (*Oncorhynchus mykiss*) would have a similar function as in mammals (63). In our study, *cyld* was up-regulated, which may indicate that in fish fed the MPLE diet this gene acted as an inhibitor of the NF-κB signaling pathway, thus acting as a negative regulator of gut inflammation (63, 64). Accordingly, the up-regulation of ubiquitin specific peptidase 4 (*usp4*) and down-regulation of Ubiquitin specific peptidase 7 (*usp7*) may be also associated to the inhibition of the activation of NF-κB and mitogen-activated protein kinases stimulated by innate immune receptor (65–67). Additionally, *chi3l1* was also up-regulated in fish fed the MPLE diet, promoting cell survival and proliferation, but not acting as a first inflammation-responsive factor. Thus, the differential regulation of the above-mentioned genes provide evidence that MPLE might induced inflammatory mechanisms related to the NF-κB signaling pathway (68, 69), which involved the production of pro-inflammatory cytokines (70). In this sense, histological data allow discarding the hypothesis that the administration of the MPLE caused an exacerbated gut inflammation, which would have been potentially detrimental for the health and condition of the intestinal mucosa of the gilthead seabream.

The Caspase‐8 is a crucial executor for apoptotic initiation in fish (71). In our study, *casp8* was significantly up-regulated in the intestine of fish fed the MPLE-supplemented diet. Several studies have reported the advantageous outcomes of having the *casp8* expressed in a controlled way, demonstrating the essential role of this gene in maintaining the gut barrier in response to mucosal pathogens by permitting inflammatory shedding and preventing necroptosis of infected cells (72, 73). In addition, *stat3* (Signal transducer and activator of transcription 3) was down-regulated, which suggests a healthy intestinal mucosa as this gene is positively regulated by pro-inflammatory cytokines under mucosal damage (74). Furthermore, our enrichment analysis showed regulation of biological processes associated with cellular proteolysis, in particular several genes coding for de- and ubiquitination (*cyld*, *uba1*, *ube2a*, *ube2g1*, *ube2d2*, *usp4*, *usp7*) and proteasome (*cops4*, *psmb2*, *psmd1*, *psmb5*, *psmd5*, *sqstm1*) that may be indirectly involved in NF-kB pathway. In addition, degradation of a protein *via* the ubiquitin-proteasome pathway and subsequent proteolysis are important mechanisms in the regulation of cell cycle, cell growth, tissue regeneration, signal transduction, and gene transcription (75).

A successful immune response involves the tight control of a wide repertoire of processes including activators and regulators at cellular and molecular level. In this context, another up-regulated gene that deserves attention is the Chitinase 3 like 1 (*chi3l1*). This gene is reputed for regulating the AKT1 signaling pathway (76), which controls innate immune cell development and function (77). Additionally, we found down-regulation of the NOD-like family CARD domain containing 3 (*nlrc3*), which plays an important role in modulating T cell responsiveness and inhibition of the pro-inflammatory mechanism (78). Besides, galectin 1 (*lgals1*) was also down-regulated in the intestine of fish fed the MPLE diet. LGALS1 is considered a master regulator of homeostatic signals to shut off T-cell effector functions (79), acting as an immunoregulator and in turn modulating the pro-inflammatory cytokines secretion (80). At receptor level, the C-type lectin domain family 4 member e (*clec4e*, also known as mincle) was down-regulated in the intestine of fish from the MPLE group. Mincle is a transmembrane germline-encoded pattern recognition receptors (PRRs) with a pivotal role in the activation of the immune response (81, 82). Thus, when considering the overall expression patterns of DEGs, the dietary-induced activation of the immune function by the tested MPLE seems to be a well-controlled process, according to the observed balance between the up- and down-regulated DEGs (18 and 14 genes, respectively) and also the functional role of the DEGs related to T cells. As mentioned above, these results are in agreement with those recently published by Salomón et al. (14) where the same feed additive showed a tightly controlled systemic immune response in an *ex vivo* assay using splenocytes stimulated by LPS.

It is also of interest to illustrate that fish fed the supplemented-MPLE diet resulted in an increase of goblet cell population in the intestinal epithelium. These secretory cells are responsible for producing a thick layer of mucus that forms the front line of innate host defense, among other functions (83). The increase in intestinal goblet cell number in fish fed MPLE-supplemented diet would benefit fish by providing an effective immune barrier against potentially pathogenic gut bacteria. In addition to changes in their number, goblet cells also changed their histochemical composition. Particularly, we observed an increment in staining intensity of neutral and acid carboxylated and sulphated glycoproteins, results that are of special relevance since O-glycosylation pattern of mucin glycoproteins and their polypeptide backbone structure in combination with the depth and viscosity of the mucus layer, are important factors that regulate gut health and condition (84). In this sense, the increase in acid carboxylated (AB pH = 2.5) and sulphated (weak and strongly ionized; AB pH = 1.0 and pH 0.5, respectively) mucin glycoproteins produced by goblet cells from fish fed the MPLE-supplemented diet would be associated to an increment in the viscous properties of the mucus (85, 86). Our results are in agreement with those reported in poultry where the administration of phytogenics from sage promoted the secretion of neutral and acidic mucins in gut as well as mucus layer thickness in the ileum (87). Regarding lectin histochemistry, mucins from fish fed the MPLE-supplemented diet showed an increase in affinity for the WGA and SBA lectins, while for SNA lectin affinity was diminished. These results indicated that the tested feed additive changed the glycosylation patterns of intestinal mucins, being of special relevance the increase in WGA lectin affinity, as they indicate an increase in the presence of sialic acid residues. Sialic acids are involved in recognition processes, and they participate in defense mechanisms against microorganisms (88). These results from lectin histochemistry reinforce the hypothesis that the MPLE-supplementation varied the composition of mucus produced by goblet cells; thus, modulating an effective immune barrier against potentially pathogenic gut bacteria.

### MPLE From Sage and Lemon Verbena Promote Gut Integrity and Cell Proliferation

The homeostatic balance between epithelial cell proliferation and apoptosis is essential for the maintenance of the epithelial function, including regulation of epithelial permeability, the inflammatory response and the absorption of nutrients (89). An imbalance in the intestinal barrier structure can trigger an uncontrollable immune reaction in the intestinal microenvironment, increasing the translocation of bacterial antigens and stimulating inflammation in the intestine (90). Thus, the maintenance of the integrity of the gut barrier is essential to counteract such imbalances and guarantee fish growth, health and welfare (91). Dietary MPLE from sage and lemon verbena modulated the expression of genes involved in biological processes related to cellular development and organization. In particular, Ras homolog family member A (*rhoa*) was up-regulated in the intestine of fish fed the MPLE-diet. RhoA belongs the small GTPase protein family, thus having a functional role in regulating many cellular events, including cell migration, organization of the cytoskeleton, cell cycle progression and cell adhesion (92). In this sense, *rhoa* expression has been associated to the assembly, regulation and maintenance of adherens (AJs) and tight (TJs) junctions (93, 94). Adherens junctions are cadherin/catenin-containing adhesive structures located below the tight junctions on the bilateral membrane (95). In particular, the Erythrocyte membrane protein band 4.1 (*epb41*) was another up-regulated gene in the intestine of fish fed the MPLE-diet. EPB41 regulates cell proliferation and adhesion (96), as well as the integrity of the AJ (95) and plays an important role in the organization and function of the TJs and AJs by establishing a link between the TJ and actin in the cytoskeleton (97). Furthermore, other DEGs related to the formation and stabilization of AJs and TJs (*f11r*, *actn4, rap1b*) were also up-regulated in the gut of fish fed the MPLE-supplemented diet. The F11 receptor (F11R) also known as the Junctional adhesion molecule (JAM)-A is a cell-cell adhesion molecule of the immunoglobulin superfamily, which is expressed by a variety of tissues, regulating diverse processes such as epithelial and endothelial barrier formation, among others (98). Our results are in agreement with those found in gilthead seabream fed a phytogenic feed additive derived from olive oil (89). Alpha-actinin-4 (ACTN4) is a member of the superfamily of actin-binding, which is localized at AJs and more specifically as a component of the *zonula occludens* of the TJ (99) and/or belt desmosomes in the *zonula adherens* (100) in connection with α-catenin, regulating the actin cytoskeleton and increasing cellular motility (101, 102). Another gene that was up-regulated by the dietary administration of MPLE from sage and lemon verbena was the *rap1b*. This gene is a member of RAS oncogene family being necessary for normal human endothelial cell function (103), as well as having a crucial role for T cell homeostasis in the intestine (104). Regarding the gut integrity barrier, *rap1b* controls epithelial permeability probably by regulation of PI3K/Akt and correct nectin localization (94).

The homoeostasis of the constantly renewing intestinal epithelium relies on an integrated control of proliferation, differentiation and apoptosis, as well as on the functional architecture of the epithelial cells. In this sense, different DEGs indicated that the MPLE-supplemented diet regulated mostly processes involved cell proliferation (*fgf18*, *pgk1*, *ldb1*, *znrf3, mtor*, *lgals1, stat3*) and de- and ubiquitination (*cyld*, *uba1*, *ube2a*, *ube2g1*, *ube2d2*, *usp4*, *usp7*) and proteasome (*cops4*, *psmb2*, *psmd1*, *psmb5*, *psmd5*, *sqstm1*) pathways. The Fibroblast growth factor 18 (FGF18) is a pleiotropic growth factor which stimulates the proliferation of mesenchymal and epithelial cells and tissues (105); thus, its up-regulation in the tissue of gilthead seabream fed the MPLE-supplemented diet suggests that the tested phytogenic feed additive might promote cell proliferation. Similar conclusions may be drawn when considering the up-regulation *ldb1* (LIM domain binding 1) and *znrf3* (Zinc and ring finger 3), since these genes regulate cell proliferation in intestinal crypts by means of the regulation of the Wnt/ß-catenin signaling (106, 107). In addition, the up-regulation of *mtor* (mammalian Target of Rapamycin) and *pgk1* (Phosphoglycerate kinase 1) indicate the regulation of intestinal cell proliferation by means of controlling cell metabolism (108, 109). These results are supported by the down-regulation of *lgals1* (galectin 1), since this gene product may act as a negative growth factor that regulates cell proliferation (110). Differential expression of genes involved in de- and ubiquitination and proteasome pathways supports the above-mentioned idea that the tested MPLE-supplemented diet promotes and regulates cell cycle associated to intestinal cell proliferation (75) as well in cell junctions (111). All these results together suggested that the MPLE from sage and lemon verbena might promote a positive effect on intestinal cell proliferation, a process that is essential for the maintenance of the integrity and health of the gastrointestinal tract (112). In line with this idea, no signs of tissue damage were observed at histological level either pathways involved in cell apoptosis were found in these animals.

## Conclusions

The present study shows that phytogenics obtained from sage and lemon verbena included at 0.1% in diets with a low FM content (7%), promoted transcriptional innate and adaptive immune responses in gut, especially through the modulation of those processes involved in T cell activation, differentiation and selection. Furthermore, the evaluated feed additive increased the number of intestinal goblet cells and modified the glycosylation properties of lectins from mucins. These changes resulted in a moderate increase in sialic acid residues, which also supports the idea that phytogenics from sage and common verbena might enhance gut immunity. Overall, this study shows that the evaluated phytogenic can be used as a safe feed additive for gilthead seabream, since its immunomodulatory properties were observed without compromising gut homeostasis and integrity of the intestinal epithelium.

## Data Availability Statement

The datasets presented in this study can be found in online repositories. The names of the repository/repositories and accession number(s) can be found in the article/**Supplementary Material**.

## Ethics Statement

The animal study was reviewed and approved by Guiding Principles for Biomedical Research Involving Animals (EU2010/63), the guidelines of the Spanish laws (law 32/2007 and RD 1201/2015), and authorized by the Ethical Committee of the Institute for Research and Technology in Food and Agriculture (IRTA, Spain).

## Author Contributions 

Conceptualization, EG. Methodology, EV-V, FER-L, CS, JO-D, RS. Formal analysis, RS, FER-L, JF, EV-V. Resources, EG, CS. Writing original draft, RS. Writing review and editing, EV-V, RS, FER-L, EG, JF, LT. Visualization, RS, FER-L, EV-V, JO-D, EG. Supervision, EG, EV-V. Project administration, EG. Funding acquisition, EG. All authors contributed to the article and approved the submitted version.

## Funding

This work has been financially supported by the project “Nutritional strategies for the improvement of productive performance: the use of functional feeds and health diets in aquaculture (DIETAplus)”, funded by JACUMAR (Ministry of Agriculture, Fisheries and Environment of Spain, MAPAMA) and FEMP (EU), as well as by the ERC (European Research Council) in MedAID project (Mediterranean Aquaculture Integrated Development; Grant Agreement Nb. 727315). FR-L thanks the support of Fondecyt Regular grant (Nb. 1211841; ANID; Government of Chile). RS is supported by a PhD grant from the government of Paraguay (BECAL). JF have been subsidized by the Industrial PhD program of Generalitat de Catalunya and TECNOVIT-FARMFAES S.L. (Nb. 2017 DI 017). EV-V was financially granted with DICYT-USACH Postdoctoral fellowship (Nb. 022043IB). Collaboration between Ibero-American researchers has been done under the framework of the network LARVAplus “Strategies for the development and improvement of fish larvae production in Ibero-America” (117RT0521) funded by the Ibero-American Program of Science and Technology for Development (CYTED, Spain).

## Conflict of Interest

JQ and JP are current NATAC BIOTECH S.L. employers. FER-L was Senior research associate by Consorcio Tecnológico de Sanidad Acuícola, Ictio Biotechnologies S.A.

The remaining authors declare that the research was conducted in the absence of any commercial or financial relationships that could be construed as a potential conflict of interest.

## References

[1] Stentiford GD Bateman IJ Hinchliffe SJ Bass D Hartnell R Santos EM. Sustainable Aquaculture Through the One Health Lens. Nat Food (2020) 1:468–74. 10.1038/s43016-020-0127-5 37128071

[2] Béné C Arthur R Norbury H Allison EH Beveridge M Bush S. Contribution of Fisheries and Aquaculture to Food Security and Poverty Reduction: Assessing the Current Evidence. World Dev (2016) 79:177–96. 10.1016/j.worlddev.2015.11.007

[3] Little DC Young JA Zhang W Newton RW Al Mamun A Murray FJ. Sustainable Intensification of Aquaculture Value Chains Between Asia and Europe: A Framework for Understanding Impacts and Challenges. Aquaculture (2018) 493:338–54. 10.1016/j.aquaculture.2017.12.033

[4] Ghamkhar R Hicks A. Comparative Environmental Impact Assessment of Aquafeed Production: Sustainability Implications of Forage Fish Meal and Oil Free Diets. Resour Conserv Recycling (2020) 161:104849. 10.1016/j.resconrec.2020.104849

[5] Stentiford GD Sritunyalucksana K Flegel TW Williams BAP Withyachumnarnkul B Itsathitphaisarn O. New Paradigms to Help Solve the Global Aquaculture Disease Crisis. PloS Pathog (2017) 13:1–6. 10.1371/journal.ppat.1006160 PMC5289612 28152043

[6] Lulijwa R Rupia EJ Alfaro AC. Antibiotic Use in Aquaculture, Policies and Regulation, Health and Environmental Risks: A Review of the Top 15 Major Producers. Rev Aquac (2020) 12:640–63. 10.1111/raq.12344

[7] Reverter M Sarter S Caruso D Avarre JC Combe M Pepey E. Aquaculture At the Crossroads of Global Warming and Antimicrobial Resistance. Nat Commun (2020) 11:1–8. 10.1038/s41467-020-15735-6 32312964 PMC7170852

[8] Asif MB Hai FI Price WE Nghiem LD. Impact of Pharmaceutically Active Compounds in Marine Environment on Aquaculture. In: Hai FI Visvanathan C Boopathy R, editors. Sustainable Aquaculture. Applied Environmental Science and Engineering for a Sustainable Future. Cham, Switzerland: Springer Cham (2018). p. 265–99. 10.1007/978-3-319-73257-2

[9] Dawood MAO Koshio S Esteban MÁ. Beneficial Roles of Feed Additives as Immunostimulants in Aquaculture: A Review. Rev Aquac (2018) 10:950–74. 10.1111/raq.12209

[10] Reyes-Cerpa S Vallejos-Vidal E Gonzalez-Bown MJ Morales-Reyes J Pérez-Stuardo D Vargas D. Effect of Yeast (*Xanthophyllomyces Dendrorhous*) and Plant (Saint John’s Wort, Lemon Balm, and Rosemary) Extract Based Functional Diets on Antioxidant and Immune Status of Atlantic Salmon (*Salmo Salar*) Subjected to Crowding Stress. Fish Shellfish Immunol (2018) 74:250–9. 10.1016/j.fsi.2017.12.061 29305990

[11] Firmino JP Vallejos-Vidal E Sarasquete C Ortiz-Delgado JB Balasch JC Tort L. Unveiling the Effect of Dietary Essential Oils Supplementation in *Sparus aurata* Gills and its Efficiency Against the Infestation by *Sparicotyle chrysophrii* . Sci Rep (2020) 10:1–23. 10.1038/s41598-020-74625-5 33082387 PMC7576129

[12] Lieke T Meinelt T Hoseinifar SH Pan B Straus DL Steinberg CEW. Sustainable Aquaculture Requires Environmental-Friendly Treatment Strategies for Fish Diseases. Rev Aquac (2020) 12:943–65. 10.1111/raq.12365

[13] Olmos-Soto J Paniagua-Michel JJ Lopez L Ochoa L. Functional Feeds in Aquaculture. In: Kim S-K, editor. Springer Handbook of Marine Biotechnology. Berlin/Heidelberg: Springer (2015). p. 1303–19. 10.1007/978-3-642-53971-8

[14] Salomón R Firmino JP Reyes-López FE Andree KB González-Silvera D Esteban MA. The Growth Promoting and Immunomodulatory Effects of a Medicinal Plant Leaf Extract Obtained From *Salvia officinalis* and *Lippia citriodora* in Gilthead Seabream (*Sparus aurata*). Aquaculture (2020) 524:735291. 10.1016/j.aquaculture.2020.735291

[15] Iwashita MKP Addo S Terhune JS. Use of Pre- and Probiotics in Finfish Aquaculture. In: Davis DA, editor. Feed Feeding Practices in Aquacualture. Sawston, UK: Woodhead Publishing (2015). p. 235–49. 10.1016/b978-0-08-100506-4.00009-x

[16] Vallejos-Vidal E Reyes-López FE Teles M MacKenzie S. The Response of Fish to Immunostimulant Diets. Fish Shellfish Immunol (2016) 56:34–69. 10.1016/j.fsi.2016.06.028 27389620

[17] Upadhaya SD Kim IH. Efficacy of Phytogenic Feed Additive on Performance, Production and Health Status of Monogastric Animals - A Review. Ann Anim Sci (2017) 17:929–48. 10.1515/aoas-2016-0079

[18] Ahmadifar E Yousefi M Karimi M Raieni RF Dadar M Yilmaz S. Benefits of Dietary Polyphenols and Polyphenol-Rich Additives to Aquatic Animal Health: An Overview. Rev Fish Sci Aquac (2020) 1–34. 10.1080/23308249.2020.1818689

[19] Celi P Cowieson AJ Fru-Nji F Steinert RE Kluenter AM Verlhac V. Gastrointestinal Functionality in Animal Nutrition and Health: New Opportunities for Sustainable Animal Production. Anim Feed Sci Technol (2017) 234:88–100. 10.1016/j.anifeedsci.2017.09.012

[20] Dawood MAO. Nutritional Immunity of Fish Intestines: Important Insights for Sustainable Aquaculture. Rev Aquac (2021) 13:642–63. 10.1111/raq.12492

[21] Hua K Cobcroft JM Cole A Condon K Jerry DR Mangott A. The Future of Aquatic Protein: Implications for Protein Sources in Aquaculture Diets. One Earth (2019) 1:316–29. 10.1016/j.oneear.2019.10.018

[22] Sitjà-Bobadilla A Peña-Llopis S Gómez-Requeni P Médale F Kaushik S Pérez-Sánchez J. Effect of Fish Meal Replacement by Plant Protein Sources on non-Specific Defence Mechanisms and Oxidative Stress in Gilthead Sea Bream (*Sparus aurata*). Aquaculture (2005) 249:387–400. 10.1016/j.aquaculture.2005.03.031

[23] Geay F Ferraresso S Zambonino-Infante JL Bargelloni L Quentel C Vandeputte M. Effects of the Total Replacement of Fish-Based Diet With Plant-Based Diet on the Hepatic Transcriptome of Two European Sea Bass (*Dicentrarchus Labrax*) Half-Sibfamilies Showing Different Growth Rates With the Plant-Based Diet. BMC Genomics (2011) 12:522. 10.1186/1471-2164-12-522 22017880 PMC3377934

[24] Gisbert E Fournier V Solovyev M Skalli A Andree KB. Diets Containing Shrimp Protein Hydrolysates Provided Protection to European Sea Bass (*Dicentrarchus Labrax*) Affected by a *Vibrio Pelagius* Natural Infection Outbreak. Aquaculture (2018) 495:136–43. 10.1016/j.aquaculture.2018.04.051

[25] Ramos-Pinto L Martos-Sitcha JA Reis B Azeredo R Fernandez-Boo S Pérez-Sánchez J. Dietary Tryptophan Supplementation Induces a Transient Immune Enhancement of Gilthead Seabream (*Sparus aurata*) Juveniles Fed Fishmeal-Free Diets. Fish Shellfish Immunol (2019) 93:240–50. 10.1016/j.fsi.2019.07.033 31310850

[26] Torrecillas S Mompel D Caballero MJ Montero D Merrifield D Rodiles A. Effect of Fishmeal and Fish Oil Replacement by Vegetable Meals and Oils on Gut Health of European Sea Bass (*Dicentrarchus Labrax*). Aquaculture (2017) 468:386–98. 10.1016/j.aquaculture.2016.11.005

[27] Estruch G Collado MC Monge-Ortiz R Vidal AT Jover-Cerdá M Peñaranda DS. Long-Term Feeding With High Plant Protein Based Diets in Gilthead Seabream (*Sparus aurata*, L.) Leads to Changes in the Inflammatory and Immune Related Gene Expression At Intestinal Level. BMC Vet Res (2018) 14:302. 10.1186/s12917-018-1626-6 30285734 PMC6171182

[28] Hoseinifar SH Doan H Dadar M Ringø E. Feed Additives, Gut Microbiota, and Health in Finfish Aquaculture. In: Derome N, editor. Microbial Communities in Aquaculture Ecosystems. Cham: Springer (2019). p. 121–42. 10.1007/978-3-030-16190-3_6

[29] Wan MLY Ling KH El-Nezami H Wang MF. Influence of Functional Food Components on Gut Health. Crit Rev Food Sci Nutr (2019) 59:1927–36. 10.1080/10408398.2018.1433629 29381385

[30] López-Nadal A Ikeda-Ohtsubo W Sipkema D Peggs D McGurk C Forlenza M. Feed, Microbiota, and Gut Immunity: Using the Zebrafish Model to Understand Fish Health. Front Immunol (2020) 11:114. 10.3389/fimmu.2020.00114 32117265 PMC7014991

[31] FAO. The State of World Fisheries and Aquaculture 2020. In: Fao Fisheries and Aquaculture Department. Rome, Italy: The Food and Agriculture Organization of the United Nations (2020).

[32] Arthur H Joubert E De Beer D Malherbe CJ Witthuhn RC. Phenylethanoid Glycosides as Major Antioxidants in *Lippia Multiflora* Herbal Infusion and Their Stability During Steam Pasteurisation of Plant Material. Food Chem (2011) 127:581–8. 10.1016/j.foodchem.2011.01.044 23140703

[33] Wójciak-Kosior M Sowa I Kocjan R Nowak R. Effect of Different Extraction Techniques on Quantification of Oleanolic and Ursolic Acid in *Lamii Albi Flos* . Ind Crops Prod (2013) 44:373–7. 10.1016/j.indcrop.2012.11.018

[34] Calduch-Giner JA Sitjà-Bobadilla A Pérez-Sánchez J. Gene Expression Profiling Reveals Functional Specialization Along the Intestinal Tract of a Carnivorous Teleostean Fish (*Dicentrarchus Labrax*). Front Physiol (2016) 7:359. 10.3389/fphys.2016.00359 27610085 PMC4997091

[35] Reyes-López FE Ibarz A Ordóñez-Grande B Vallejos-Vidal E Andree KB Balasch JC. Skin Multi-Omics-Based Interactome Analysis: Integrating the Tissue and Mucus Exuded Layer for a Comprehensive Understanding of the Teleost Mucosa Functionality as Model of Study. Front Immunol (2021) 11:613824. 10.3389/fimmu.2020.613824 33613538 PMC7890662

[36] Szklarczyk D Gable AL Lyon D Junge A Wyder S Huerta-Cepas J. STRING V11: Protein-protein Association Networks With Increased Coverage, Supporting Functional Discovery in Genome-Wide Experimental Datasets. Nucleic Acids Res (2019) 47:607–13. 10.1093/nar/gky1131 PMC6323986 30476243

[37] Stelzer G Rosen N Plaschkes I Zimmerman S Twik M Fishilevich S. The GeneCards Suite: From Gene Data Mining to Disease Genome Sequence Analyses. Curr Protoc Bioinf (2016) 54:1.30.1–1.30.33. 10.1002/cpbi.5 27322403

[38] Uniprot. Uniprot: A Worldwide Hub of Protein Knowledge. Nucleic Acids Res (2019) 47:506–15. 10.1093/nar/gky1049 PMC6323992 30395287

[39] Sarasquete C Gisbert E Ribeiro L Vieira L Dinis MT. Glyconjugates in Epidermal, Branchial and Digestive Mucous Cells and Gastric Glands of Gilthead Sea Bream, *Sparus aurata*, Senegal Sole, *Solea Senegalensis* and Siberian Sturgeon, *Acipenser Baeri* Development. Eur J Histochem (2001) 45:267–78. 10.4081/1637 11759813

[40] Pearse AGE. Histochemistry: Theoretical and Applied. Vol. 2, Analytical Technology. J Pathol (1985) 147:234–4. 10.1002/path.1711470319

[41] Sarasquete C Cárdenas S González de Canales ML Pascual E. Oogenesis in the Bluefin Tuna, *Thunnus Thynnus* L.: A Histological and Histochemical Study. Histol Histopathol (2002) 17:775–88. 10.14670/HH-17.775 12168787

[42] Yamamoto T Kawai K Oshima S. Distribution of Mocous Cells on the Body Surface of Japanase Flounder *Paralichthys Olivaceus* . J Fish Biol (2011) 78:848–59. 10.1111/j.1095-8649.2010.02898.x. 21366577

[43] Parra D Reyes-Lopez FE Tort L. Mucosal Immunity and B Cells in Teleosts: Effect of Vaccination and Stress. Front Immunol (2015) 6:354. 10.3389/fimmu.2015.00354 26236311 PMC4502357

[44] Estensoro I Ballester-Lozano G Benedito-Palos L Grammes F Martos-Sitcha JA Mydland LT. Dietary Butyrate Helps to Restore the Intestinal Status of a Marine Teleost (*Sparus aurata*) Fed Extreme Diets Low in Fish Meal and Fish Oil. PloS One (2016) 11:1–21. 10.1371/journal.pone.0166564 PMC5127657 27898676

[45] Caturla N Funes L Perez-Fons L Micol V. A Randomized, Double-Blinded, Placebo Controlled Study of the Effect of a Combination of Lemon Verbena Extract and Fish Oil Promega-3 Fatty Acid on Joint Management. J Altern Complement Med (2011) 17:1051–63. 10.1089/acm.2010.0410 PMC3221510 22087615

[46] Alipieva K Korkina L Orhan IE Georgiev MI. Verbascoside - a Review of its Occurrence, (Bio)Synthesis and Pharmacological Significance. Biotechnol Adv (2014) 32:1065–76. 10.1016/j.biotechadv.2014.07.001 25048704

[47] Ghorbani A Esmaeilizadeh M. Pharmacological Properties of *Salvia Officinalis* and its Components. J Tradit Complement Med (2017) 7:433–40. 10.1016/j.jtcme.2016.12.014 PMC5634728 29034191

[48] Secombes CJ Wang T. The Innate and Adaptive Immune System of Fish. In: Austin B, editor. Infectious Disease in Aquaculture: Prevention and Control. Sawston, UK: Woodhead Publishing (2012). p. 3–68. 10.1533/9780857095732.1.3

[49] Nakanishi T Shibasaki Y Matsuura Y. T Cells in Fish. Biology (2015) 4:640–63. 10.3390/biology4040640 PMC4690012 26426066

[50] Scapigliati G Fausto AM Picchietti S. Fish Lymphocytes: An Evolutionary Equivalent of Mammalian Innate-Like Lymphocytes? Front Immunol (2018) 9:971. 10.3389/fimmu.2018.00971 29867952 PMC5949566

[51] Rombout JHWM Abelli L Picchietti S Scapigliati G Kiron V. Teleost Intestinal Immunology. Fish Shellfish Immunol (2011) 31:616–26. 10.1016/j.fsi.2010.09.001 20832474

[52] Fischer U Utke K Somamoto T Köllner B Ototake M Nakanishi T. Cytotoxic Activities of Fish Leucocytes. Fish Shellfish Immunol (2006) 20:209–26. 10.1016/j.fsi.2005.03.013 15939625

[53] Salinas I Parra D. Fish Mucosal Immunity: Intestine. In: Beck BH Peatman E, editors. Mucosal Health in Aquaculture. London, UK: Academic Press (2015). p. 135–70. 10.1016/B978-0-12-417186-2.00006-6

[54] Ma H Tao W Zhu S. T Lymphocytes in the Intestinal Mucosa: Defense and Tolerance. Cell Mol Immunol (2019) 16:216–24. 10.1038/s41423-019-0208-2 PMC6460495 30787416

[55] Weichhart T Haidinger M Katholnig K Kopecky C Poglitsch M Lassnig C. Inhibition of mTOR Blocks the Anti-Inflammatory Effects of Glucocorticoids in Myeloid Immune Cells. Blood (2011) 117:4273–83. 10.1182/blood-2010-09-310888 21368289

[56] Laplante M Sabatini D. mTOR Signaling in Growth Control and Disease. Cell (2012) 149:274–93. 10.1016/j.cell.2012.03.017.mTOR PMC3331679 22500797

[57] Abuhagr AM MacLea KS Chang ES Mykles DL. Mechanistic Target of Rapamycin (mTOR) Signaling Genes in Decapod Crustaceans: Cloning and Tissue Expression of Mtor, Akt, Rheb, and P70 S6 Kinase in the Green Crab, *Carcinus Maenas*, and Blackback Land Crab, *Gecarcinus Lateralis* . Comp Biochem Physiol (2014) 168A:25–39. 10.1016/j.cbpa.2013.11.008 24269559

[58] Säemann MD Haidinger M Hecking M Hörl WH Weichhart T. The Multifunctional Role of mTOR in Innate Immunity: Implications for Transplant Immunity. Am J Transplant (2009) 9:2655–61. 10.1111/j.1600-6143.2009.02832.x 19788500

[59] Yang H Wang X Zhang Y Liu H Liao J Shao K. Modulation of TSC-mTOR Signaling on Immune Cells in Immunity and Autoimmunity. J Cell Physiol (2014) 229:17–26. 10.1002/jcp.24426 23804073

[60] Karki R Malireddi R Zhu Q Kanneganti TD. NLRC3 Regulates Cellular Proliferation and Apoptosis to Attenuate the Development of Colorectal Cancer. Cell Cycle (2017) 16:1243–51. 10.1080/15384101.2017.1317414 PMC5531621 28598238

[61] Dorrington MG Fraser IDC. Nf-κb Signaling in Macrophages: Dynamics, Crosstalk, and Signal Integration. Front Immunol (2019) 10:705. 10.3389/fimmu.2019.00705 31024544 PMC6465568

[62] Pistol GC Marin DE Rotar MC Ropota M Taranu I. Bioactive Compounds From Dietary Whole Grape Seed Meal Improved Colonic Inflammation Via Inhibition of MAPKs and NF-kB Signaling in Pigs With DSS Induced Colitis. J Funct Foods (2020) 66:103708. 10.1016/j.jff.2019.103708

[63] Jang JH Lee HM Kim H Cho JH. Molecular Cloning and Functional Analysis of Deubiquitinase CYLD in Rainbow Trout, *Oncorhynchus Mykiss* . Fish Shellfish Immunol (2020) 101:135–42. 10.1016/j.fsi.2020.03.058 32224281

[64] Sun SC. Cyld: A Tumor Suppressor Deubiquitinase Regulating NF-B Activation and Diverse Biological Processes. Cell Death Differ (2010) 17:25–34. 10.1038/cdd.2009.43 19373246 PMC5848464

[65] Zhang J Stirling B Temmerman ST Ma CA Fuss IJ Derry JM. Impaired Regulation of NF-kappaB and Increased Susceptibility to Colitis-Associated Tumorigenesis in CYLD-deficient Mice. J Clin Invest (2006) 116:3042–9. 10.1172/JCI28746 PMC1616194 17053834

[66] Colleran A Collins PE O’Carroll C Ahmed A Mao X McManus B. Deubiquitination of NF-κb by Ubiquitin-Specific Protease-7 Promotes Transcription. Proc Natl Acad Sci USA (2013) 110:618–23. 10.1073/pnas.1208446110 PMC3545798 23267096

[67] Li Z Hao Q Luo J. USP4 Inhibits p53 and NF-κb Through Deubiquitinating and Stabilizing HDAC2. Oncogene (2016) 35:2902–12. 10.1038/onc.2015.349 PMC4895393 26411366

[68] Regula KM Ens K Kirshenbaum LA. IKK Beta is Required for Bcl-2-mediated NF-Kappa B Activation in Ventricular Myocytes. J Biol Chem (2002) 277:38676–82. 10.1074/jbc.M206175200 12167626

[69] Chang MX Xiong F Wu XM Hu YW. The Expanding and Function of NLRC3 or NLRC3-like in Teleost Fish: Recent Advances and Novel Insights. Dev Comp Immunol (2021) 114:103859. 10.1016/j.dci.2020.103859 32896535

[70] El Aidy S Derrien M Aardema R Hooiveld G Richards SE Dane A. Transient Inflammatory-Like State and Microbial Dysbiosis are Pivotal in Establishment of Mucosal Homeostasis During Colonisation of Germ-Free Mice. Benef Microbes (2014) 5:67–77. 10.3920/BM2013.0018 24322881

[71] dos Santos N Vale A Reis M Silva M. Fish and Apoptosis: Molecules and Pathways. Curr Pharm Design (2008) 14:148–69. 10.2174/138161208783378743 18220827

[72] Günther C Buchen B He GW Hornef M Torow N Neumann H. Caspase-8 Controls the Gut Response to Microbial Challenges by Tnf-α-Dependent and Independent Pathways. BMJ J Gut (2015) 64:601–10. 10.1136/gutjnl-2014-307226 PMC4392221 25379949

[73] Sun S Ge X Zhu J Zhang W Zhang Q. Molecular Cloning, Immunohistochemical Localization, Characterization and Expression Analysis of Caspase-8 From the Blunt Snout Bream (*Megalobrama Amblycephala*) Exposed to Ammonia. Fish Shellfish Immunol (2015) 47:645–54. 10.1016/j.fsi.2015.10.016 26492992

[74] Neufert C Pickert G Zheng Y Wittkopf N Warntjen M Nikolaev A. Activation of Epithelial STAT3 Regulates Intestinal Homeostasis. Cell Cycle (2010) 9:652–5. 10.4161/cc.9.4.10615 20160497

[75] Glickman MH Ciechanover A. The Ubiquitin-Proteasome Proteolytic Pathway: Destruction for the Sake of Construction. Physiol Rev (2002) 82:373–428. 10.1152/physrev.00027.2001 11917093

[76] Chen CC Llado V Eurich K Tran HT Mizoguchi E. Carbohydrate-Binding Motif in Chitinase 3-Like 1 (CHI3L1/YKL-40) Specifically Activates Akt Signaling Pathway in Colonic Epithelial Cells. Clin Immunol (2011) 140:268–75. 10.1016/j.clim.2011.04.007 PMC3154968 21546314

[77] Zhang Y Wang X Yang H Liu H Lu Y Han L. Kinase AKT Controls Innate Immune Cell Development and Function. Immunology (2013) 140:143–52. 10.1111/imm.12123 PMC3784161 23692658

[78] Paria A Deepika A Sreedharan K Makesh M Chaudhari A Purushothaman CS. Identification of Nod Like Receptor C3 (NLRC3) in Asian Seabass, *Lates Calcarifer*: Characterisation, Ontogeny and Expression Analysis After Experimental Infection and Ligand Stimulation. Fish Shellfish Immunol (2016) 55:602–12. 10.1016/j.fsi.2016.06.029 27346158

[79] Rabinovich GA Toscano MA. Turning “Sweet” on Immunity: Galectin-glycan Interactions in Immune Tolerance and Inflammation. Nat Rev Immunol (2009) 9:338–52. 10.1038/nri2536 19365409

[80] Rabinovich GA Gruppi A. Galectins as Immunoregulators During Infectious Processes: From Microbial Invasion to the Resolution of the Disease. Parasite Immunol (2005) 27:103–14. 10.1111/j.1365-3024.2005.00749.x 15910418

[81] Richardson MB Williams SJ. MCL and Mincle: C-Type Lectin Receptors That Sense Damaged Self and Pathogen-Associated Molecular Patterns. Front Immunol (2014) 5:288. 10.3389/fimmu.2014.00288 25002863 PMC4066366

[82] Clément M Basatemur G Masters L Baker L Bruneval P Iwawaki T. Necrotic Cell Sensor Clec4e Promotes a Proatherogenic Macrophage Phenotype Through Activation of the Unfolded Protein Response. Circulation (2016) 134:1039–51. 10.1161/CIRCULATIONAHA.116.022668 27587433

[83] Cornick S Tawiah A Chadee K. Roles and Regulation of the Mucus Barrier in the Gut. Tissue Barriers (2015) 3:e982426. 10.4161/21688370.2014.982426 25838985 PMC4372027

[84] Huang JY Lee SM Mazmanian SK. The Human Commensal Bacteroides Fragilis Binds Intestinal Mucin. Anaerobe (2011) 17:137–41. 10.1016/j.anaerobe.2011.05.017 PMC3163789 21664470

[85] Kumari U Yashpal M Mittal S Mittal AK. Histochemical Analysis of Glycoproteins in the Secretory Cells in the Gill Epithelium of a Catfish, *Rita Rita* (Siluriformes, Bagridae). Tissue Cell (2009) 41:271–80. 10.1016/j.tice.2008.12.006 19233444

[86] Díaz AO García AM Escalante AH Goldemberg AL. Glycoproteins Histochemistry of the Gills of *Odontesthes Bonariensis* (Teleostei, Atherinopsidae). J Fish Biol (2010) 77:1665–73. 10.1111/j.1095-8649.2010.02803.x 21078025

[87] Čapkovičová A Maková Z Piešová E Alves A Faix Š Faixová Z. Evaluation of the Effects of *Salvia Officinalis* Essential Oil on Plasma Biochemistry, Gut Mucus and Quantity of Acidic and Neutral Mucins in the Chicken Gut. Acta Vet (2014) 64:138–48. 10.2478/acve-2014-0014

[88] Traving C Schauer R. Structure, Function and Metabolism of Sialic Acids. Cell Mol Life Sci (1998) 54:1330–49. 10.1007/s000180050258 PMC7082800 9893709

[89] Gisbert E Andree KB Quintela JC Calduch-Giner JA Ipharraguerre IR Pérez-Sánchez J. Olive Oil Bioactive Compounds Increase Body Weight, and Improve Gut Health and Integrity in Gilthead Sea Bream (*Sparus aurata*). Br J Nutr (2017) 117:351–63. 10.1017/S0007114517000228 28245885

[90] Chelakkot C Ghim J Ryu SH. Mechanisms Regulating Intestinal Barrier Integrity and its Pathological Implications. Exp Mol Med (2018) 50:103. 10.1038/s12276-018-0126-x PMC6095905 30115904

[91] Cain K Swan C. Barrier Function and Immunology. In: Grosell M Farrell AP Brauner CJ, editors. Fish Physiology. London, UK: Academic Press (2011). p. 111–34. 10.1016/S1546-5098(10)03003-7

[92] Marjoram RJ Lessey EC Burridge K. Regulation of RhoA Activity by Adhesion Molecules and Mechanotransduction. Curr Mol Med (2014) 14:199–208. 10.2174/1566524014666140128104541 24467208 PMC3929014

[93] Terry S Nie M Matter K Balda MS. Rho Signaling and Tight Junction Functions. Physiology (2010) 25:16–26. 10.1152/physiol.00034.2009 20134025

[94] Citalán-Madrid AF García-Ponce A Vargas-Robles H Betanzos A Schnoor M. Small GTPases of the Ras Superfamily Regulate Intestinal Epithelial Homeostasis and Barrier Function Via Common and Unique Mechanisms. Tissue Barriers (2013) 1-5:e26938. 10.4161/tisb.26938 PMC3942330 24868497

[95] Yang S Guo X Debnath G Mohandas N An X. Protein 4.1R Links E-Cadherin/β-Catenin Complex to the Cytoskeleton Through its Direct Interaction With β-Catenin and Modulates Adherens Junction Integrity. Biochim Biophys Acta (BBA) - Biomembr (2009) 1788:1458–65. 10.1016/j.bbamem.2009.03.022 PMC4409867 19376086

[96] Zhang J Yang S An C Wang J Yan H Huang Y. Comprehensive Characterization of Protein 4.1 Expression in Epithelium of Large Intestine. Histochem Cell Biol (2014) 142:529–39. 10.1007/s00418-014-1224-z 24912669

[97] Mattagajasingh SN Huang SC Hartenstein JS Benz EJ. Characterization of the Interaction Between Protein 4.1R and ZO-2: A Possible Link Between the Tight Junction and the Actin Cytoskeleton. J Biol Chem (2000) 275:30573–85. 10.1074/jbc.M004578200 10874042

[98] Ebnet K. Junctional Adhesion Molecules (Jams): Cell Adhesion Receptors With Pleiotropic Functions in Cell Physiology and Development. Physiol Rev (2017) 97:1529–54. 10.1152/physrev.00004.2017 28931565

[99] Chen VC Li X Perreault H Nagy JI. Interaction of Zonula Occludens-1 (ZO-1) With α-Actinin-4: Application of Functional Proteomics for Identification of PDZ Domain-Associated Proteins. J Proteome Res (2006) 5:2123–34. 10.1021/pr060216l 16944923

[100] Milanini J Fayad R Partisani M Lecine P Borg JP Franco M. EFA6 Proteins Regulate Lumen Formation Through α-Actinin 1. J Cell Sci (2018) 131:1–15. 10.1242/jcs.209361 29246944

[101] Knudsen KA Soler AP Johnson KR Wheelock MJ. Interaction of α-Actinin With the Cadherin/Catenin Cell-Cell Adhesion Complex Via α-Catenin. J Cell Biol (1995) 130:67–77. 10.1083/jcb.130.1.67 7790378 PMC2120515

[102] Honda K Yamada T Endo R Ino Y Gotoh M Tsuda H. Actinin-4, a Novel Actin-Bundling Protein Associated With Cell Motility and Cancer Invasion. J Cell Biol (1998) 140:1383–93. 10.1083/jcb.140.6.1383 PMC2132673 9508771

[103] Yan J Li F Ingram DA Quilliam LA. Rap1a Is a Key Regulator of Fibroblast Growth Factor 2-Induced Angiogenesis and Together With Rap1b Controls Human Endothelial Cell Functions. Mol Cell Biol (2008) 28:5803–10. 10.1128/mcb.00393-08 PMC2546931 18625726

[104] Ishihara S Nishikimi A Umemoto E Miyasaka M Saegusa M Katagiri K. Dual Functions of Rap1 are Crucial for T-cell Homeostasis and Prevention of Spontaneous Colitis. Nat Commun (2015) 6:1–15. 10.1038/ncomms9982 PMC4686857 26634692

[105] Haque T Nakada S Hamdy RC. A Review of FGF18: its Expression, Signaling Pathways and Possible Functions During Embryogenesis and Post-Natal Development. Histol Histopathol (2007) 1:97–105. 10.14670/HH-22.97 17128416

[106] Dey-Guha I Mukhopadhyay M Phillips M Westphal H. Role of Ldb1 in Adult Intestinal Homeostasis. Int J Biol Sci (2009) 5:686–94. 10.7150/ijbs.5.686 PMC2777272 19918297

[107] Spit M Koo BK Maurice MM. Tales From the Crypt: Intestinal Niche Signals in Tissue Renewal, Plasticity and Cancer. Open Biol (2018) 8:180120. 10.1098/rsob.180120 30209039 PMC6170508

[108] Nie H Ju H Fan J Shi X Cheng Y Cang X. O-GlcNAcylation of PGK1 Coordinates Glycolysis and TCA Cycle to Promote Tumor Growth. Nat Commun (2020) 11:36. 10.1038/s41467-019-13601-8 31911580 PMC6946671

[109] Fritsch SD Weichhart T. Metabolic and Immunologic Control of Intestinal Cell Function by Mtor. Int Immunol (2020) 32:455–65. 10.1093/intimm/dxaa015 PMC7617511 32140726

[110] Sundblad V Quintar AA Morosi LG Niveloni SI Cabanne A Smecuol E. Galectins in Intestinal Inflammation: Galectin-1 Expression Delineates Response to Treatment in Celiac Disease Patients. Front Immunol (2018) 9:379. 10.3389/fimmu.2018.00379 29545799 PMC5837985

[111] Cai J Culley MK Zhao Y Zhao J. The Role of Ubiquitination and Deubiquitination in the Regulation of Cell Junctions. Protein Cell (2018) 9:754–69. 10.1007/s13238-017-0486-3 PMC6107491 29080116

[112] Wong WM Wright NA. Cell Proliferation in Gastrointestinal Mucosa. J Clin Pathol (1999) 52:321–33. 10.1136/jcp.52.5.321 PMC1023065 10560350

